# Equine arteritis virus long-term persistence is orchestrated by CD8^+^ T lymphocyte transcription factors, inhibitory receptors, and the CXCL16/CXCR6 axis

**DOI:** 10.1371/journal.ppat.1007950

**Published:** 2019-07-29

**Authors:** Mariano Carossino, Pouya Dini, Theodore S. Kalbfleisch, Alan T. Loynachan, Igor F. Canisso, R. Frank Cook, Peter J. Timoney, Udeni B. R. Balasuriya

**Affiliations:** 1 Louisiana Animal Disease Diagnostic Laboratory and Department of Pathobiological Sciences, School of Veterinary Medicine, Louisiana State University, Baton Rouge, LA, United States of America; 2 Maxwell H. Gluck Equine Research Center, Department of Veterinary Science, University of Kentucky, Lexington, KY, United States of America; 3 Faculty of Veterinary Medicine, Ghent University, Merelbeke, Belgium; 4 Department of Biochemistry and Molecular Genetics, School of Medicine, University of Louisville, Louisville, KY, United States of America; 5 University of Kentucky Veterinary Diagnostic Laboratory, Department of Veterinary Science, University of Kentucky, Lexington, KY, United States of America; 6 Department of Veterinary Clinical Medicine, and Department of Comparative Biosciences, College of Veterinary Medicine, University of Illinois Urbana-Champaign, Urbana, IL, United States of America; Freie Universitat Berlin, GERMANY

## Abstract

Equine arteritis virus (EAV) has the unique ability to establish long-term persistent infection in the reproductive tract of stallions and be sexually transmitted. Previous studies showed that long-term persistent infection is associated with a specific allele of the *CXCL16* gene (*CXCL16S*) and that persistence is maintained despite the presence of local inflammatory and humoral and mucosal antibody responses. Here, we performed transcriptomic analysis of the ampullae, the primary site of EAV persistence in long-term EAV carrier stallions, to understand the molecular signatures of viral persistence. We demonstrated that the local CD8^+^ T lymphocyte response is predominantly orchestrated by the transcription factors eomesodermin (EOMES) and nuclear factor of activated T-cells cytoplasmic 2 (NFATC2), which is likely modulated by the upregulation of inhibitory receptors. Most importantly, EAV persistence is associated with an enhanced expression of *CXCL16* and *CXCR6* by infiltrating lymphocytes, providing evidence of the implication of this chemokine axis in the pathogenesis of persistent EAV infection in the stallion reproductive tract. Furthermore, we have established a link between the *CXCL16* genotype and the gene expression profile in the ampullae of the stallion reproductive tract. Specifically, *CXCL16* acts as a “hub” gene likely driving a specific transcriptional network. The findings herein are novel and strongly suggest that RNA viruses such as EAV could exploit the CXCL16/CXCR6 axis in order to modulate local inflammatory and immune responses in the male reproductive tract by inducing a dysfunctional CD8^+^ T lymphocyte response and unique lymphocyte homing in the reproductive tract.

## Author summary

A distinctive feature of equine arteritis virus (EAV) is its ability to establish long-term persistent infection in the stallion reproductive tract in the presence of strong immune and inflammatory responses. The data presented herein provides insight into the molecular signature of the inflammatory response during persistent infection in the male reproductive tract, and shows that long-term persistence is associated with the predominance of specific CD8^+^ T lymphocyte transcription factors that drive the inflammatory process in the reproductive tract, along with the upregulation of inhibitory receptors and CXCL16/CXCR6, a chemokine axis strongly implicated in EAV persistence. Furthermore, the host’s *CXCL16* genotype drives the changes in transcriptional factors that favors EAV persistent infection. These findings have a broad translational importance in the immunopathogenesis of EAV and other persistent viral infections in the male reproductive tract of animals and humans, as well as in the prevention and treatment of such infections.

## Introduction

Equine arteritis virus (EAV) is a positive-sense, single-stranded RNA virus that belongs to the family *Arteriviridae*, order *Nidovirales* [1]. EAV is the causative agent of equine viral arteritis (EVA), an economically important systemic, reproductive and respiratory disease of equids [2–8]. Transmission of EAV can occur through the respiratory or venereal routes by acutely infected horses or solely through the venereal route by persistently infected stallions [4, 8–10]. EAV infection in horses can be either asymptomatic or associated with a wide range of clinical signs, including dependent edema, conjunctivitis, periorbital or supraorbital edema, respiratory distress, urticaria and leukopenia [2–4, 8, 11–18]. Infection of pregnant mares can result in abortion or birth of congenitally infected foals that frequently develop a fatal bronchointerstitial pneumonia or pneumoenteric syndrome [19]. Most importantly, EAV can establish long-term persistent infection in the reproductive tract of stallions (carrier state) resulting in continuous shedding of infectious virus in their semen [2–4], which guarantees the perpetuation of the virus in equine populations [2–4, 7–11, 20, 21]. EAV persistent infection is testosterone-dependent [22] and can last from several weeks or months (i.e., virus shedding in semen ≤ 1 year following infection [short-term carrier]) to years or even life-long (i.e., virus shedding in semen >1 year following infection [long-term carrier]). Furthermore, persistently infected stallions do not exhibit clinical signs of disease or impairment of fertility [4, 8–10, 18, 20, 21, 23–25]. To date, the immunopathogenesis of persistent EAV infection in the reproductive tract of the stallion is not fully elucidated and is currently under investigation in our laboratory.

Recently, it has been shown that the outcome of EAV infection in the stallion is dependent on host genetic factors, clearly associated with a specific allele of the *CXCL16* gene (*CXCL16S*) that encodes for the C-X-C motif chemokine ligand 16 (CXCL16). Importantly, it has been demonstrated that CXCL16S acts as a cellular receptor for EAV while CXCL16R does not [26]. Furthermore, it has also been shown that EAV has a specific tropism for a subset of CD8^+^ T and CD21^+^ B lymphocytes and stromal cells primarily in the ampullae and to a lesser extent in the other accessory sex glands (vesicular, prostate and bulbourethral glands) of persistently infected stallions [23, 25, 27–29]. Moreover, EAV persists in the male genital tract despite the presence of strong inflammatory (mediated mainly by CD8^+^ T lymphocytes) and EAV-specific humoral and mucosal antibody responses [23, 24]. Also, it has been recently demonstrated that EAV long-term persistent infection is associated with the specific downregulation of microRNA (miRNA) eca-mir-128 in seminal exosomes along with an enhanced expression of CXCL16 in the ampullae, a putative target of eca-mir-128, at the site of persistent infection [30]. Understanding the mechanisms of EAV persistence in the stallion reproductive tract is critical to the success of efforts to develop novel therapeutics for elimination of the carrier state. Thus, the long-term goal of our studies is to specifically identify the mechanism(s) of EAV persistent infection in the stallion reproductive tract. In this study, we hypothesized that persistent EAV infection induces a specific immunological milieu in the stallion reproductive tract that favors viral immune evasion by modulating the host’s local immune and inflammatory responses at the site of viral persistence, a process driven by specific transcription factors and the CXCL16/CXCR6 chemokine axis.

## Results

### Establishment of persistent infection in EAV experimentally infected stallions

The clinical outcome and establishment of EAV persistent infection after intranasal challenge with EAV KY84 strain in this group of stallions (n = 8) has been previously described [18, 23, 24]. Of the 8 infected stallions, six stopped shedding in <1 year post-infection and were classified as short-term carrier stallions (Table 1). Conversely, two of the 8 stallions continued to shed EAV in their semen for >1 year post-infection and were classified as long-term carrier stallions (Table 1). A naturally infected, long-term carrier stallion (stallion E) was also included in the study as previously described [23]. All long-term carrier stallions carried the *CXCL16S* allele (Table 1). All experimentally infected stallions were humanely euthanized at 726 days post-infection (dpi) and tissues collected for analysis.

**Table 1:** EAV experimentally and naturally infected stallions used in this study (n = 12).

Stallion ID	Carrier status	CXCL16 genotype	Susceptible CD3^+^ T lymphocytes (%)	Duration of viral shedding (dpi)
**L136**	**Long-term**	***CXCL16S/CXCL16S***	**5.32**	**≥726**
L137	Short-term	*CXCL16R/CXCL16R*	0.31	149
L138	Short-term	*CXCL16S/CXCL16R*	3.16	128
L139	Short-term	*CXCL16R/CXCL16R*	0.49	380
**L140**	**Long-term**	***CXCL16S/CXCL16R***	**3.75**	**≥726**
L141	Short-term	*CXCL16S/CXCL16S*	8.87	380
L142	Short-term	*CXCL16R/CXCL16R*	0.6	198
L143	Short-term	*CXCL16R/CXCL16R*	0.48	ND
**E**	**Long-term**	***CXCL16S/CXCL16R***	**9.49**	**~7 years**
N121	Naïve	*CXCL16R/CXCL16R*	0.09	NA
O103	Naïve	*CXCL16R/CXCL16R*	0.08	NA
O113	Naïve	*CXCL16S/CXCL16R*	2.25	NA

Short-term carrier stallions: duration of viral shedding in semen approximately ≤1 year; long-term carrier stallions: duration of viral shedding in semen >1 year (indicated in bold). NA, not applicable; and ND, not determined.

Susceptible CD3^+^ T lymphocytes (%) reflects the percentage of peripheral blood CD3^+^ T lymphocytes susceptible to EAV infection *in vitro*. The presence of this susceptible population is strongly associated with the *CXCL16S* allele [27–29, 31].

For behavioral reasons, semen collection from L143 was not feasible, and the carrier status could not be determined until the end of the study. Absence of infective virus, viral antigen, and viral RNA from tissues derived from the reproductive tract after euthanasia and the minimal inflammation observed in the ampullae and other accessory sex glands in addition to the homozygosity for the *CXCL16R* allele were indicative that this animal stopped shedding earlier in the course of the study and was considered to be a short-term carrier stallion [23, 24].

### Global transcriptome analysis of the ampullae, the primary site of EAV persistence in stallions

**Fig 1:**
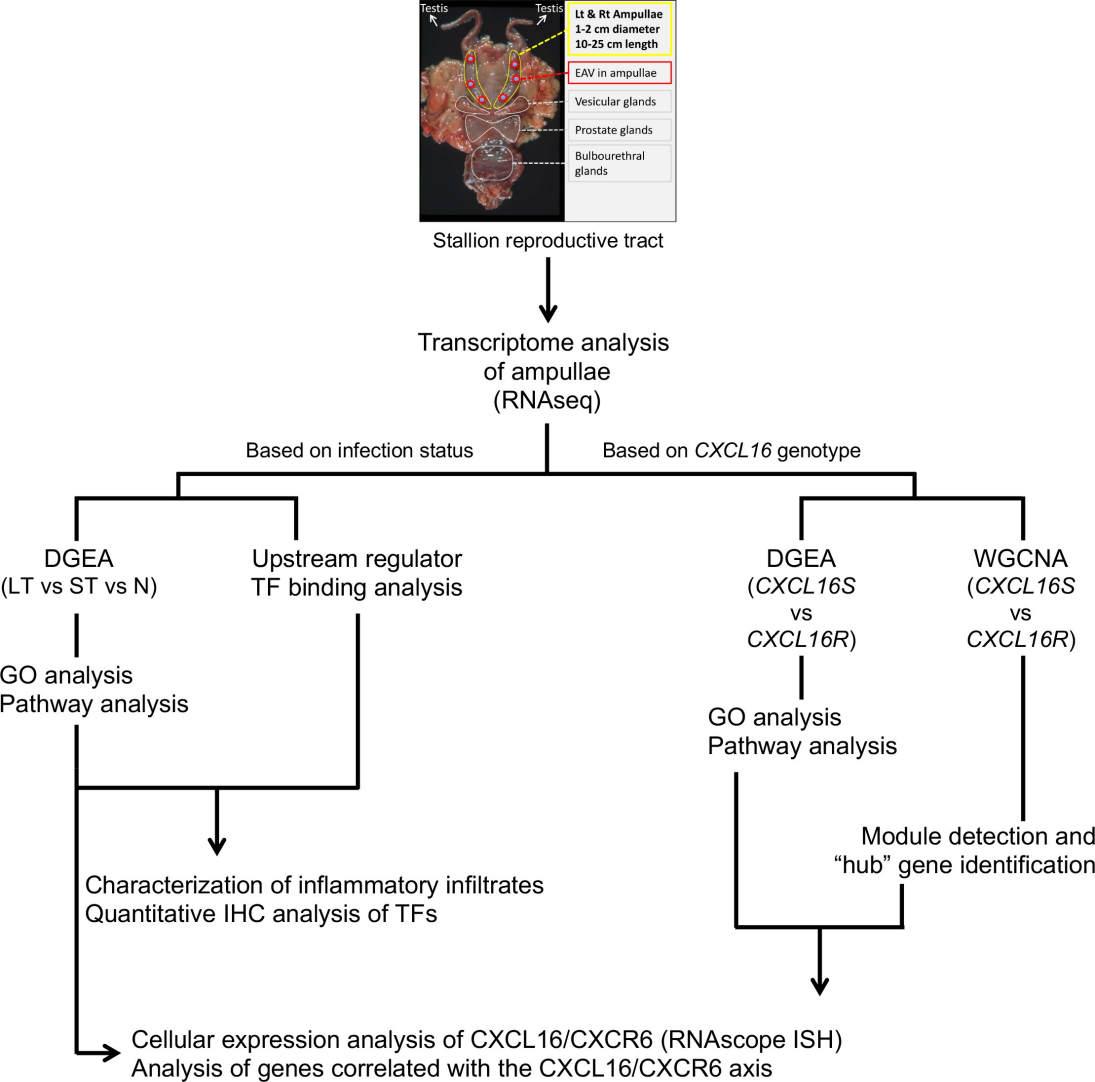
Analysis workflow carried out in this study. Transcriptome analysis of the ampullae (RNAseq) was performed along with subsequent extensive GO and pathway analysis, IHC and ISH. EAV, equine arteritis virus; DGEA, differential gene expression analysis; WGCNA, weighted gene co-expression network analysis; LT, EAV long-term carrier stallion; ST, EAV short-term carrier stallion; N, naïve stallion; TF, transcription factor; GO, gene ontology; IHC, immunohistochemistry; ISH, *in situ* hybridization.

**Fig 2:**
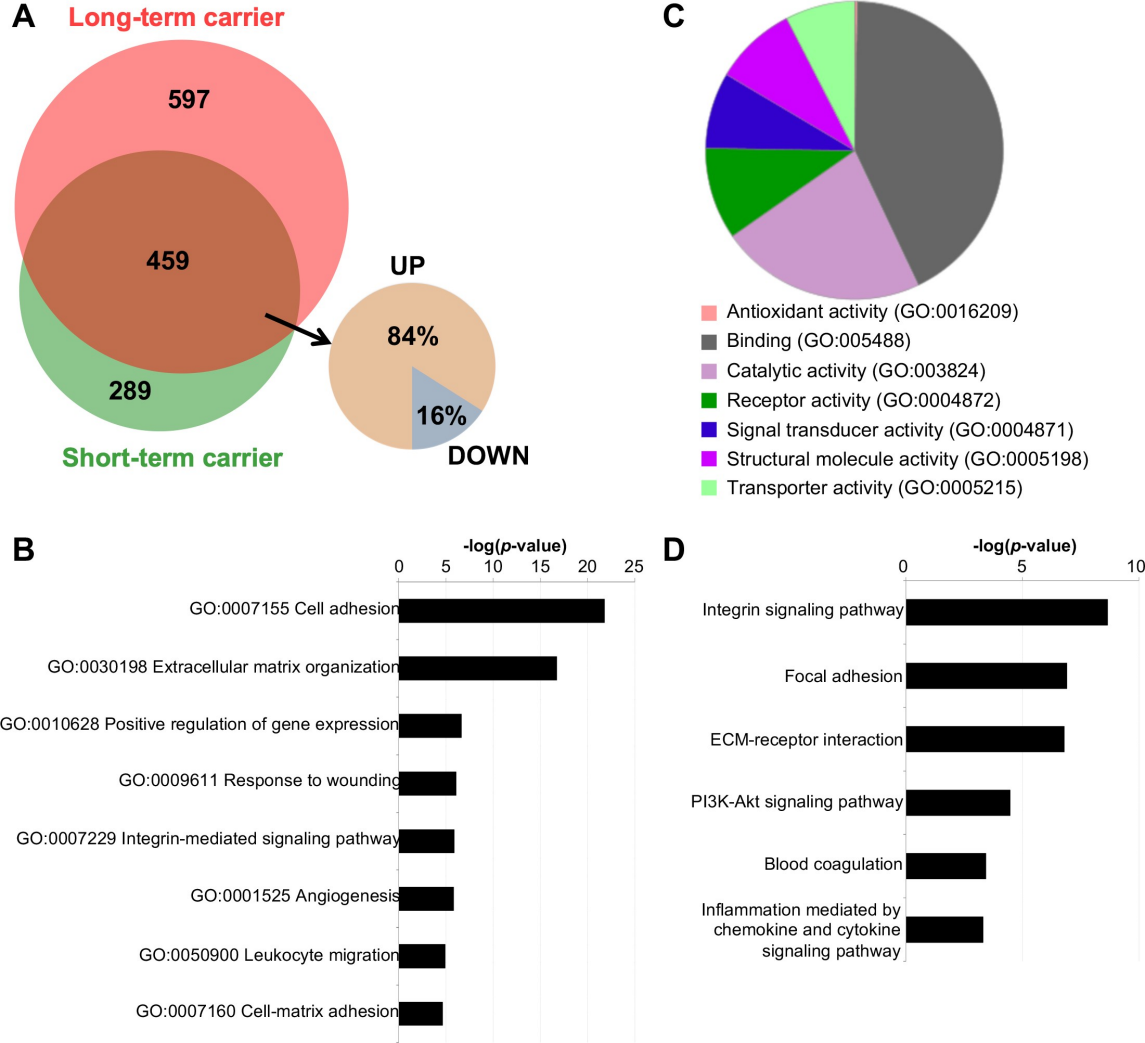
Differential gene expression profile in the ampullae of long-term (n = 3) and short-term (n = 6) EAV carrier stallions compared to the naïve group (n = 3). (A) The Venn diagram depicts the differentially expressed genes (DEGs) common between long-term and short-term carrier stallions, with a clear majority being upregulated. (B and C) GO analysis (biological process and molecular function) of commonly upregulated genes between long-term and short-term carrier stallions reveal involvement in biological processes associated with cell adhesion, extracellular matrix organization, response to wounding, integrin signaling, among others, with a high proportion of genes presenting binding and catalytic activities. (D) Significant pathways associated with commonly upregulated genes.

In order to obtain a comprehensive understanding of the molecular basis of the host response mechanisms to EAV at the site of persistence, we performed comparative whole transcriptome analysis of the ampullae from naïve (n = 3), long-term (n = 3) and short-term (n = 6) carrier stallions collected at the end of the study (726 dpi). The analysis workflow for this study can be depicted in Fig 1. A total of 1,056 and 748 differentially expressed genes (DEGs, false discovery rate [FDR] < 0.1 and log_2_ fold-change > 1 and < -1) were identified in long-term and short-term carrier stallions compared to the naïve group (n = 3), respectively. Among the DEGs observed in long-term carrier stallions, a total of 896 genes were found to be upregulated (log_2_ fold-change > 1 over the naïve group, 84.8%) while 160 genes were downregulated (log_2_ fold-change < -1 over the naïve group, 15.2%). Similarly, a clear majority of the DEGs in short-term carrier stallions were upregulated (647 genes, 86.5%) while a total of 101 genes were downregulated (13.5%). Furthermore, 459 common DEGs were identified when comparing both long-term and short-term carrier stallions to the naïve group. Among these, 386 genes (84%) were upregulated while 73 genes (16%) were downregulated (Fig 2A). Functional annotation analysis of commonly upregulated DEGs was performed using DAVID and PANTHER bioinformatics tools [32, 33], and demonstrated that these were mainly involved in biological processes associated with adhesion (cell adhesion, extracellular matrix organization, integrin-mediated signaling, leukocyte migration and cell-matrix adhesion; Fig 2B). Regarding their molecular function, commonly upregulated genes were significantly associated with protein binding (heparin, integrin, collagen, extracellular matrix and actin binding; Fig 2C). Relevant biological pathways involving commonly upregulated genes are depicted in Fig 2D. Pathway analysis using Ingenuity Pathway Analysis (IPA, Qiagen, Valencia, CA) identified similar pathways involved including granulocyte/agranulocyte adhesion and diapedesis, integrin signaling, protein kinase signaling and epithelial adherens junction signaling, among others (S1 Table). No statistically significant gene ontology (GO) terms for biological processes or molecular functions were obtained for common downregulated genes.

**Fig 3:**
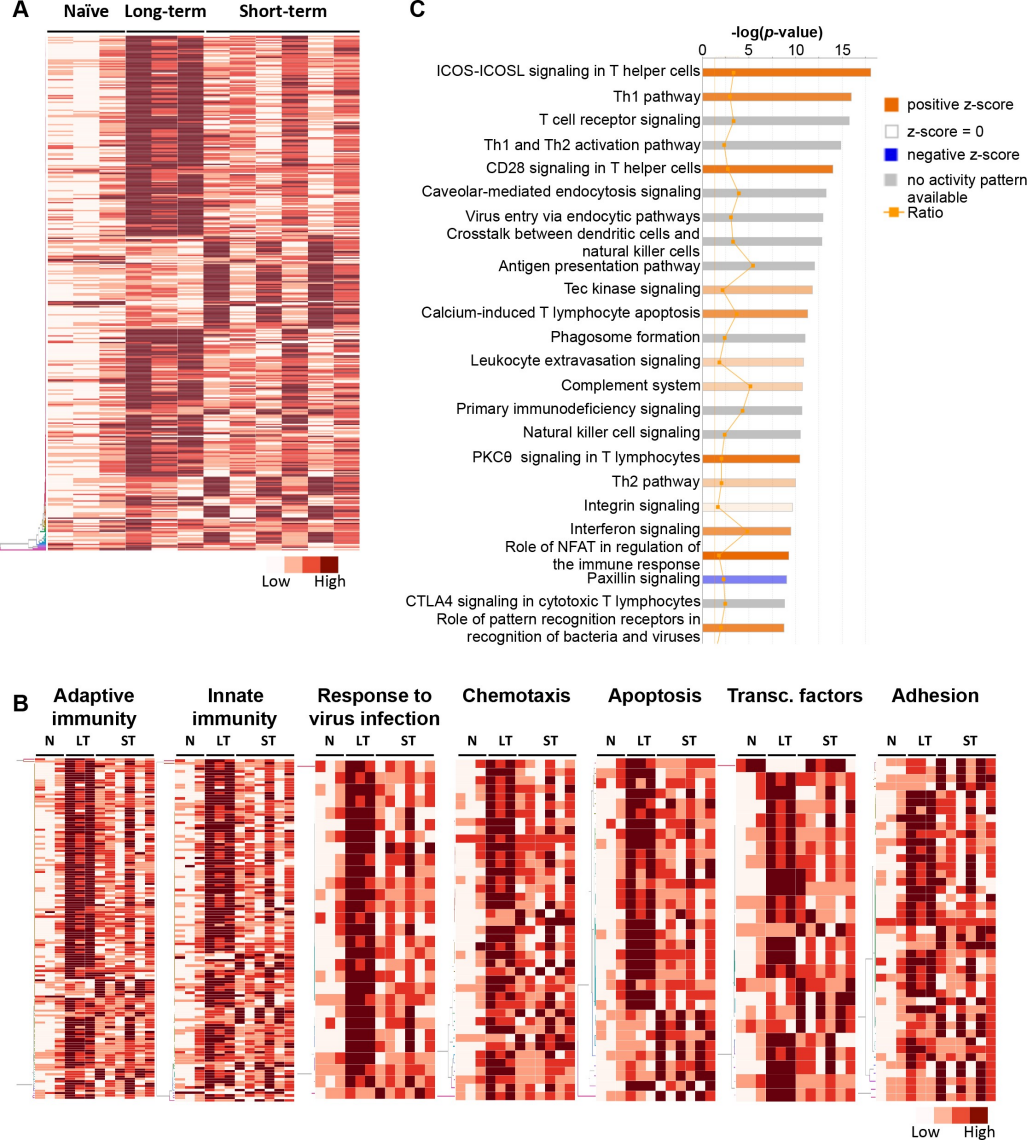
Differential gene expression profile in the ampullae of long-term EAV carrier stallions (n = 3) reveal significant differences compared to short-term carrier stallions (n = 6). (A) Heatmap depicting the expression pattern of the differentially expressed genes (DEGs) between long-term and short-term carrier stallions (n = 390 genes). The majority of the DEGs between these two groups were upregulated. Darker reds are indicative of a higher expression. The interactive heatmap can be found here (B) Representative heatmaps showing changes in gene expression levels during long and short-term viral persistence. DEGs (n = 390) were classified based on their Gene Ontology (GO) terms and selected categories are depicted. Differential gene expression analysis revealed a significant upregulation of genes involved in adaptive (the interactive heatmap can be found here) and innate (the interactive heatmap can be found here) immune responses, response to virus infection (the interactive heatmap can be found here), chemotaxis (the interactive heatmap can be found here), apoptosis (the interactive heatmap can be found here) and adhesion (the interactive heatmap can be found here). In addition, perturbation in the gene expression of several transcription factors was observed (the interactive heatmap can be found here). Darker reds are indicative of a higher expression. N, naïve; LT, long-term carrier stallions; ST, short-term carrier stallions. (C) Top 25 canonical pathways associated with the DEGs (n = 390) observed between long-term and short-term carrier stallions as determined by Ingenuity Pathway Analysis (IPA). Canonical pathways were predominantly associated with T lymphocyte pathways and signaling mechanisms.

For the identification of specific molecular signatures in the inflammatory response during long-term EAV persistence in the ampullae, we additionally performed differential gene expression analysis between long-term and short-term carrier stallions. Comparative whole transcriptome analysis demonstrated that 390 genes were differentially expressed between these two groups, with a high proportion of genes being upregulated in long-term compared to short-term carrier stallions (284 genes [72.8%]; Fig 3A). DEGs were categorized based on selected GO terms (biological process; S1 Fig) and their expression patterns in long-term, short-term carrier and naïve stallions are depicted in Fig 3B. There was a clear upregulation of genes associated with all these biological processes in long-term carrier stallions (Fig 3B). Additional upregulated genes associated with other immune-related biological processes (e.g. effector functions, antigen processing and presentation, sensing/signaling/transcriptional regulation, among others) are shown in Table 2, some of which presented a strong upregulation (fold change > 2). In the case of downregulated genes, muscle contraction, structural constituent of muscle and actin binding were identified as the only significant biological process and molecular functions, respectively.

**Table 2:** Grouping of selected DEGs out of 284 upregulated genes observed in long-term carrier (n = 3) compared to short-term carrier stallions (n = 6).

Gene symbol	Gene name	Fold change*
***Effector function***		
FASLG	Fas ligand	3.78
GZMA/GZMK	Granzyme A/Granzyme K	3.62/2.83
KLRK1	Killer cell lectin-like receptor K1	3.33
LYZ	Lysozyme	4.21
MX1/MX2	Interferon-induced GTP-binding protein MX1/MX2	1.45/2.70
NLRC3, 4, 5	NOD-like receptor family CARD domain containing 3,4,5	2.12
OAS2	2’-5’-oligoadenylate synthetase 2	1.26
OASL	2’-5’-oligoadenylate synthetase like	3.05
CD7	CD7 molecule	2.60
***Antigen processing and presentation***		
TAP1	Transporter associated with antigen processing 1	
HLA-DMA (class II)	HLA class II histocompatibility antigen, DM alpha chain	3.29
HLA-DQA (class II)	HLA class II histocompatibility antigen, DQ alpha chain	4.27
HLA-DQB2 (class II)	HLA class II histocompatibility antigen, DQ beta chain 2	2.98
HLA-DRA (class II)	HLA class II histocompatibility antigen, DR alpha chain	3.30
HLA-DRB (class II)	HLA class II histocompatibility antigen, DR beta chain	2.47
HLA-DQB (class II)	HLA class II histocompatibility antigen, DQ beta chain	2.65
CD74 (class II)	CD74 molecule	2.53
HLA-E (class I)	HLA class I histocompatibility antigen, E alpha chain	1.64
EQMHCB2 (class I)	*Equus caballus* MHC class I heavy chain	1.70
EQMHCC1 (class I)	*Equus caballus* MHC class I heavy chain	1.83
***Cytokines*, *chemokines and cytokine receptors***		
IL7R	Interleukin 7 receptor	2.06
IL16	Interleukin 16	2.69
IL21	Interleukin 21	NA
CCL2	C-C motif chemokine ligand 2 (MCP1)	4.93
CCL5	C-C motif chemokine ligand 5 (RANTES)	3.31
CXCL9	C-X-C motif chemokine ligand 9	5.92
CXCL10	C-X-C motif chemokine ligand 10	4.75
CXCL11	C-X-C motif chemokine ligand 11	5.38
CXCL13	C-X-C motif chemokine ligand 13	NA
CXCL16	C-X-C motif chemokine ligand 16	1.84
IL2RG	Interleukin 2 receptor subunit gamma	2.63
IL10RA	Interleukin 10 receptor subunit alpha	2.75
IL18RAP	Interleukin 18 receptor accessory protein	2.82
***Adhesion***		
CD6	CD6 molecule	2.25
CD53	CD53 molecule	3.22
CD226	CD226 molecule	2.89
CXCL16	C-X-C motif chemokine ligand 16	1.84
SIGLEC1	Sialic acid binding Ig-like lectin 1	2.97
LY9	Lymphocyte antigen 9	2.47
***T lymphocyte immune synapse***		
CD3D, CD3E, CD3G	CD3 delta, epsilon, gamma chain	2.93
CD4	CD4 molecule	3.35
CD8A	CD8 molecule, alpha chain	3.26
CD40LG	CD40 ligand	2.41
CD45	CD45 molecule	1.84
ITK	Interleukin 2 inducible T-cell kinase	2.36
LAT	Linker for activation of T cells	3.19
LCK	Lymphocyte protein tyrosine kinase	2.47
THEMIS	Thymocyte selection associated protein	2.86
ZAP70	Zeta chain T cell receptor associated protein kinase 70	3.04
***Sensing/signaling/transcription factors***		
BATF	Basic leucine zipper transcription factor	3.83
CD180	CD180 molecule	3.01
CIITA	Class II major histocompatibility complex transactivator	2.69
EOMES	Eomesodermin	3.08
IKZF3	Ikaros family zinc finger 3	3.39
IRF1	Interferon regulatory factor 1	1.41
IRF4	Interferon regulatory factor 4	4.41
IRF9	Interferon regulatory factor 9	1.65
NFATC2	Nuclear factor of activated T cells 2	2.49
PRDM1	PR domain zinc finger protein 1 (BLIMP-1)	2.61
STAT1	Signal transducer and activator of transcription 1	1.97
STAT2	Signal transducer and activator of transcription 2	1.03
STAT4	Signal transducer and activator of transcription 4	1.99
TLR7/TLR8	Toll-like receptor 7/8	2.21/4.35
TBX21	T-box transcription factor TBX21 (T-bet)	1.99
***Complement***		
C1QA,B,C	Complement C1q subcomponent subunit A,B,C	3.52
C1R/C1S	Complement C1r/C1s	1.61/1.96
C2	Complement C2	2.27
C3AR1	Complement C3a receptor 1	2.28
***Caspases***		
CASP4	Caspase 4	1.98
CASP12	Caspase 12	3.29

*log_2_ over naïve group. NA, not applicable since FPKM = 0 in naïve group

Pathway analysis using IPA identified that the DEGs observed between long-term and short-term carrier stallions were primarily involved in several T-lymphocyte associated canonical pathways (Fig 3C). Among the top 25 canonical pathways identified, nine were predictively activated (z score ≥ 2) in long-term carrier stallions and, interestingly, these included the type 1 T helper lymphocyte (Th1) pathway, interferon signaling, regulation of the immune response by nuclear factor of activated T-cells (NFAT), and cytotoxic T lymphocyte-associated protein 4 (CTLA-4) signaling in cytotoxic T lymphocytes. Other significant canonical pathways involved in T lymphocyte and natural killer cell-mediated responses were also observed, although activation predictions could not be determined (Fig 3C). In summary, a clear majority of the DEGs in long-term carrier stallions demonstrated to be upregulated, with specific involvement in adaptive (specifically T lymphocyte-associated) and innate immune responses, including pathways related to the regulation of the immune response.

### Characterization of the inflammatory response in the ampullae of EAV long-term carrier stallions

**Fig 4:**
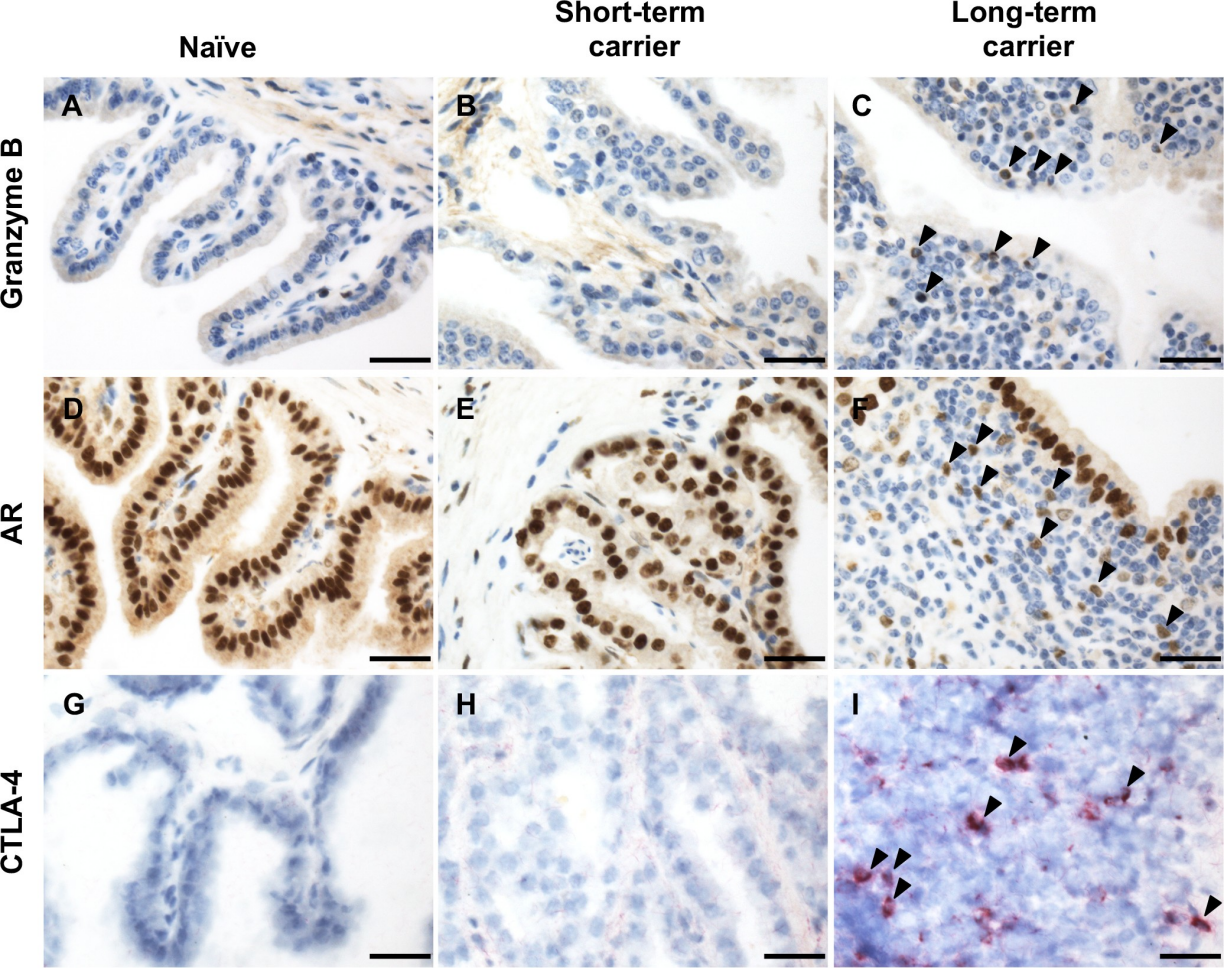
Further characterization of the inflammatory response at the site of persistence during EAV long-term persistent infection. (A-C) Presence of scattered granzyme B^+^ cells (C, arrowheads) in inflammatory infiltrates of long-term carrier stallions. (D-F) The nuclear expression of AR was predominant in the glandular epithelia and scattered stromal cells regardless of infection status. Interestingly, cells within inflammatory infiltrates showed AR expression (F, arrowheads). Granzyme B and AR-specific immunostaining. DAB. 400X. Bar = 20 μm. (G-I) Inflammatory infiltrates in long-term carrier stallions presented scattered CTLA-4^+^ T lymphocytes (I, arrowheads). CTLA-4-specific immunostaining. Fast Red. 400X. Bar = 20 μm.

The inflammatory response to EAV during long-term persistent infection was characterized both histologically and immunohistochemically (IHC). Histopathological examination of the ampullae from long-term carrier stallions showed a moderate to severe, multifocal lymphoplasmacytic ampullitis (S2 Fig). Interestingly, the inflammatory infiltrates were characterized by extensive numbers of CD8^+^ T lymphocytes, particularly in the lamina propria of the luminal villi along with their intra- and sub-epithelial localization (S2 Fig). The inflammatory response was also characterized by lower numbers of CD4^+^ T lymphocytes [23] and a significantly higher number of mononuclear cells expressing granzyme B in long-term compared to short-term carrier stallions (*p*-value = 0.0141, Fig 4A–4C). Since maintenance of EAV long-term persistent infection is testosterone-dependent, immunohistochemical (IHC) evaluation of the androgen receptor (AR) was undertaken to assess the cellular expression within the ampulla. IHC analysis identified its widespread nuclear expression in glandular epithelia and stromal cells in all stallions, and inflammatory (mononuclear) cell infiltrates in long-term and short-term carrier stallions (Fig 4D–4F). Interestingly, a significantly higher number of AR^+^ cells were found within inflammatory infiltrates of long-term compared to short-term carrier stallions (*p*-value = 0.0237). In addition, lymphocyte and epithelial cell proliferation were evaluated by Ki-67 immunostaining [34], which demonstrated that neither of these cell types was actively proliferating in any of the experimental groups.

**Fig 5:**
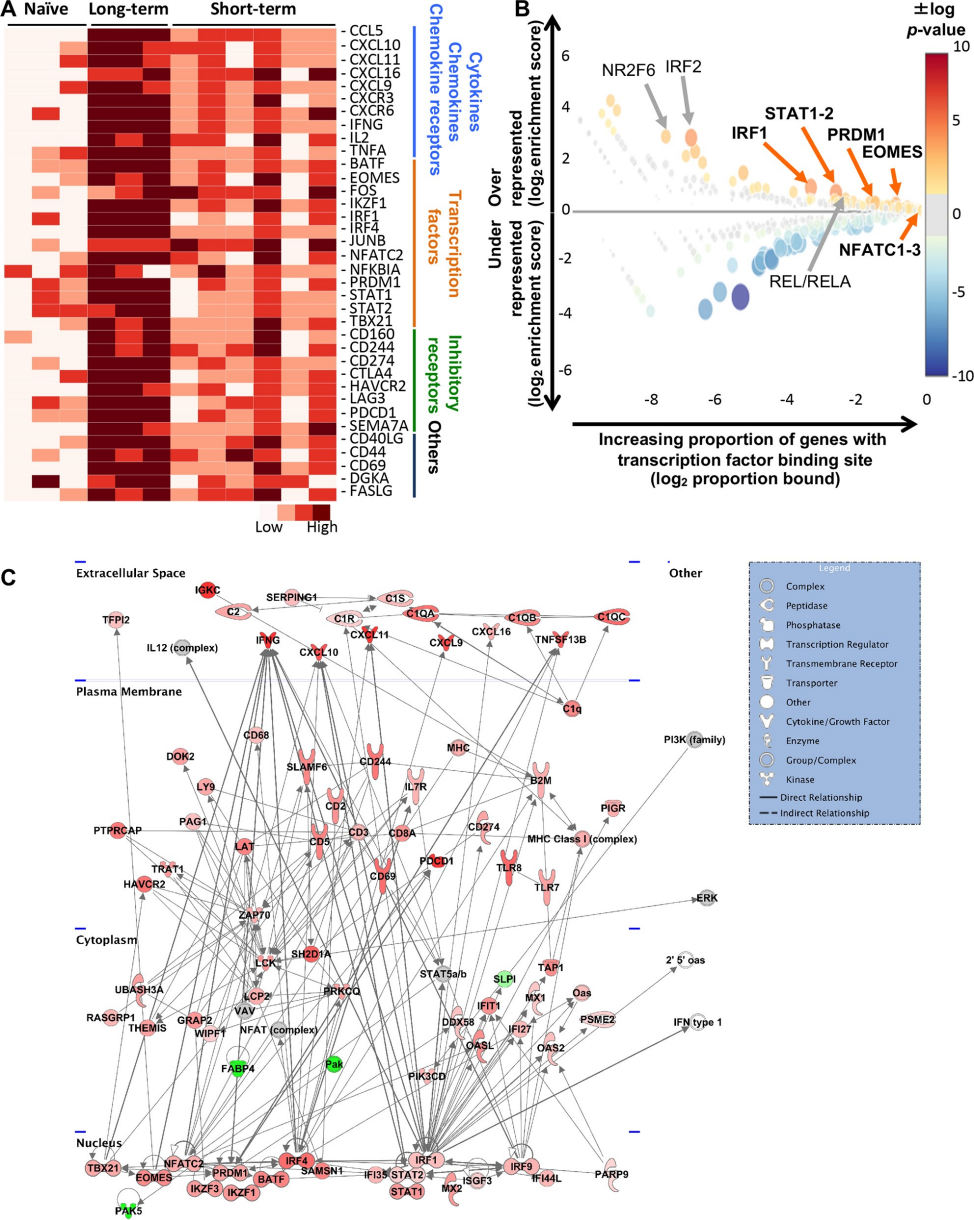
Gene expression analysis of selected genes, transcription factor enrichment analysis and molecular networks associated with long-term EAV persistence. (A) Heatmap depicting gene expression profiling by RT-qPCR of selected genes. Upregulation of specific chemokines/cytokines and chemokine receptors (including *CXCL16* and *CXCR6*), selected transcription factors and inhibitory receptors was observed. The heatmap was generated using -ΔCt values. (B) Analysis of putative transcription factor site enrichment in DEGs between long-term and short-term carrier stallions using CiiiDER. Color and size of circles reflect *p*-value of enrichment. Over-represented transcription factors of potential interest are depicted. Those differentially expressed in long-term carrier stallions are depicted in bold. (C) Molecular network associated with DEGs observed between long-term and short-term carrier stallions. The network is driven by specific transcription factors, some of which were over-represented as determined by transcription factor binding site enrichment analysis. Upregulated and downregulated genes (log_2_ fold-change compared to short-term carrier stallions) are depicted in red and green color, respectively. The degree in color intensity reflects the magnitude of the fold-change, where intense red or green indicate a higher or lower fold-change, respectively. Only direct relationships are shown.

In terms of the chemokine and cytokine profile associated with the persistent inflammatory response, RNAseq analysis identified the upregulation of the T lymphocyte-associated C-C motif chemokine ligand 2 and 5 (*CCL2* and *CCL5*), as well as a subset of related C-X-C motif chemokine ligands including *CXCL9*, *CXCL10* and *CXCL11*. Relative gene expression analysis by RT-qPCR demonstrated that *CCL5*, *CXCL9* and *CXCL10* were significantly upregulated in long-term compared to short-term carrier and naïve stallions (*p*-values < 0.0001) as well as in short-term carrier compared to the naïve group (*p*-value = 0.0253). *CXCL11* was uniquely upregulated in long-term carrier (*p*-values < 0.0001) and not statistically different between short-term carrier and naïve stallions (*p*-value > 0.05, Fig 5A). Similarly, the expression of *CXCR3*, the common chemokine receptor for CXCL9, CXCL10 and CXCL11, was significantly higher in long-term carrier stallions (*p*-values < 0.0001, Fig 5A). These findings suggest a specific role of C-X-C motif chemokines in the homing of lymphocytes into the reproductive tract during long-term persistence. Furthermore, RT-qPCR analysis of interferon gamma (*IFNG*), tumor necrosis factor alpha (*TNFA*) and interleukin 2 (*IL2*) demonstrated that both *IFNG* and *TNFA* were strongly upregulated in long-term carrier stallions (*p*-values < 0.0001, Fig 5A), which could be associated with activation of the Th1 pathway (see below). However, no statistically significant differences in the relative expression of *IL2* were observed between long-term and short-term carrier stallions (*p*-value > 0.05). Taken together, EAV persistent infection is mainly associated with the infiltration of CD8^+^ T lymphocytes and granzyme B^+^ cells with lower numbers of CD4^+^ T lymphocytes. In addition, EAV persistence is associated with the upregulation of C-X-C homing chemokines (C-X-C motif chemokine ligands) and their specific receptors (C-X-C motif chemokine receptors), and limited lymphocyte proliferation at the site of persistence.

### Transcription factor profile of inflammatory infiltrates during EAV long-term persistent infection in stallions

In order to further understand the molecular elements that drive the inflammatory response during EAV long-term persistence, we analyzed the expression of transcription factors (TFs) combined with transcription factor binding, upstream regulator and molecular network analysis. Differential gene expression analysis identified a subset of TFs that were differentially upregulated in long-term compared to short-term carrier and naïve stallions (FDR < 0.1 and log_2_ fold-change over naïve group >1, Table 2, Fig 3C). In order to understand the biological significance of these TFs in the regulation of the DEGs identified between long-term and short-term carrier stallions (390 genes), examination of TF usage was undertaken. For this purpose, we performed upstream regulator analysis on IPA (filtering solely for nucleic acid-binding molecules/TFs) and identified a subset of significant TFs (n = 16) acting as upstream regulators for the DEG dataset (S2 Table). Noteworthy, these included interferon regulatory factors (*IRF1*, *IRF4*, *IRF9*), signal transducer and activator of transcription 1 and 4 (*STAT1* and *STAT4*), nuclear factor of activated T-cells cytoplasmic 2 (*NFATC2*), T-box transcription factor TBX21 (*TBX21* or T-bet), PR domain zinc finger protein 1 (*PRDM1*, also known as transcriptional repressor B lymphocyte-induced maturation protein-1/BLIMP-1), eomesodermin (*EOMES*) and IKAROS family zinc finger 1 (*IKZF1*), among others. These results were complimented by TF binding site analysis using CiiiDER analysis tool (Centre for Innate Immunity and Infectious Diseases, Hudson Institute of Medical Research, Victoria, Australia) [35]. Transcription factor enrichment analysis demonstrated that several TFs were significantly over-represented in long-term carrier stallions including *IRF1*, *IRF2*, *STAT1-2*, *PRDM1*, *EOMES*, *NFATC1*, *NFATC2*, *NFATC3*, basic leucine zipper ATF-like transcription factor (*BATF*), proto-oncogene c-Rel (*REL*) and nuclear factor NF-kappa-B p65 subunit (*RELA*) (Fig 5B). The TFs identified and differentially expressed in long-term carrier stallions were selected for further analysis. These included *IRF1*, *IRF4*, *STAT1*, *STAT2*, *TBX21*, *BATF*, *PRDM1*, *EOMES* and *NFATC2*, and were involved in four immune-related molecular networks from the top 10 molecular networks identified by IPA (cell-mediated immune response, antimicrobial/inflammatory response, humoral immune response and immunological disease, Fig 5C). RT-qPCR analysis of the selected TFs was performed to confirm their relative expression (Fig 5A). While both long-term and short-term carrier stallions presented higher levels of expression of *BATF*, *EOMES*, *IRF4* and *TBX21* compared to the naïve group (*p*-values ≤ 0.0004 and *p*-values ≤ 0.0398, respectively), long-term carrier stallions showed a significantly higher upregulation of these transcripts compared to the short-term carrier group (*p*-values ≤ 0.0004). Interestingly, *IRF1*, *NFATC2*, *PRDM1*, *STAT1* and *STAT2* demonstrated to be solely upregulated in the long-term carrier group (*p*-values ≤ 0.0045), and no significant differences were observed in their relative expression between short-term carrier and naïve stallions (*p*-values > 0.1636; Fig 5A). Thus, this suggests that these TFs could act as the specific drivers of the local inflammatory/immune response during persistent infection.

**Fig 6:**
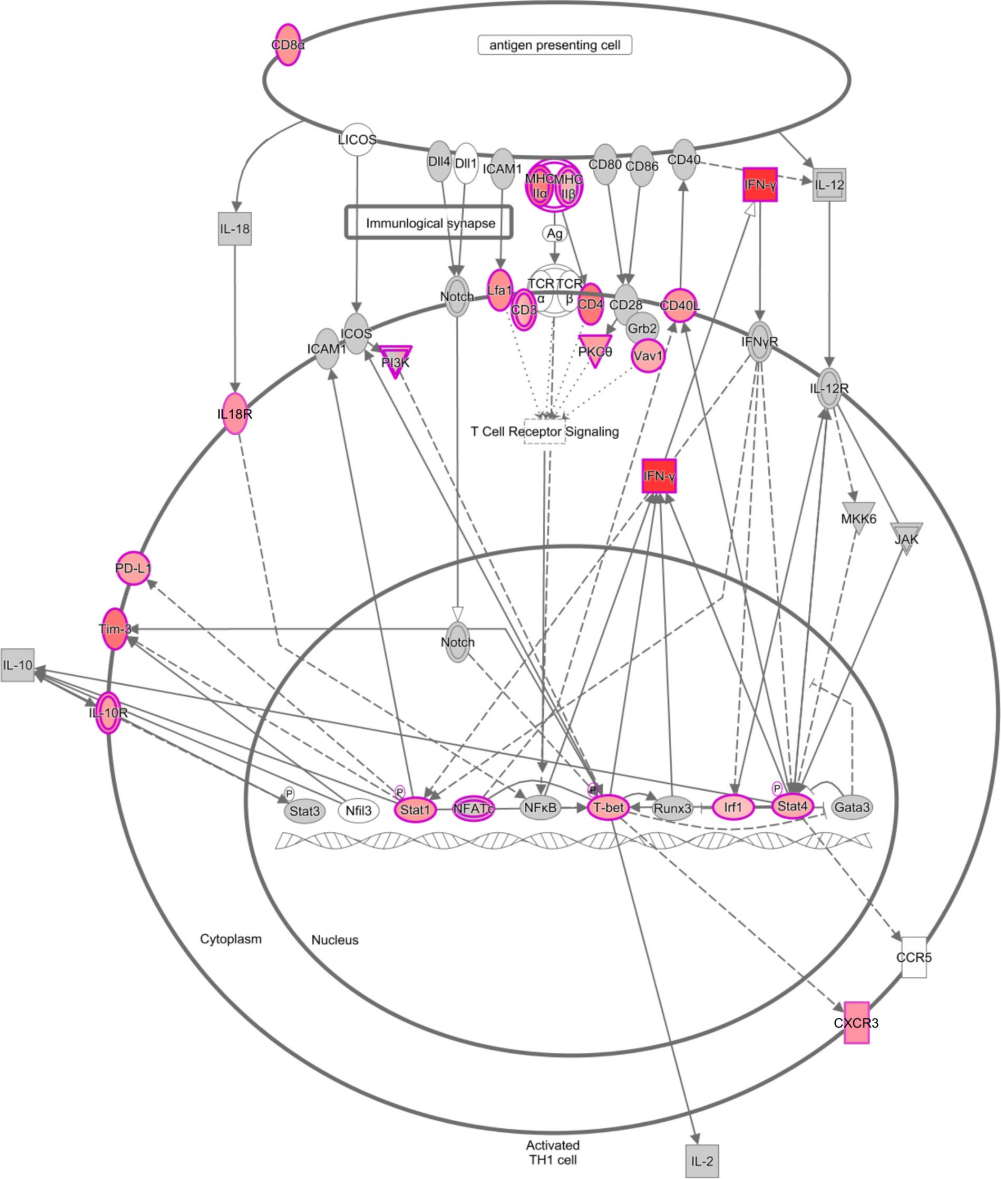
T-helper 1 (Th1) pathway analysis based on DEGs observed between long-term and short-term carrier stallions. Numerous transcription factor genes associated with the Th1 pathway are upregulated in long-term persistently infected stallions along with other related genes including cytokines. Upregulated genes (log_2_ fold-change compared to short-term carrier stallions) are depicted in red. Non-differentially expressed genes within the pathway are depicted in grey. The degree in color intensity reflects the magnitude of the fold-change, where intense red indicates a higher fold-change. Direct (solid arrows) and indirect (dashed arrows) relationships are shown.

Interestingly, transcriptomic analysis via RNAseq and subsequent RT-qPCR confirmation also demonstrated the upregulation of a subset of genes associated with the Th1 differentiation process during long-term persistence. These specifically included Th1-specific TFs *STAT1*, *STAT4*, and *TBX21* (master Th1 transcription factor). In contrast, no relative expression differences in Th2-specific TFs GATA binding protein 3 (*GATA3;* master Th2 transcription factor) and STAT6 were observed (Table 2 and Fig 6). Overall, the combined use of differential gene expression, TF binding, upstream regulator and molecular network analyses identified a group of TFs strongly involved in the regulation of the gene expression profile at the site of EAV persistent infection, including cytotoxic and Th1-mediated responses.

### EOMES and NFATC2 constitute the predominant transcription factors in inflammatory infiltrates during long-term EAV persistence

**Fig 7:**
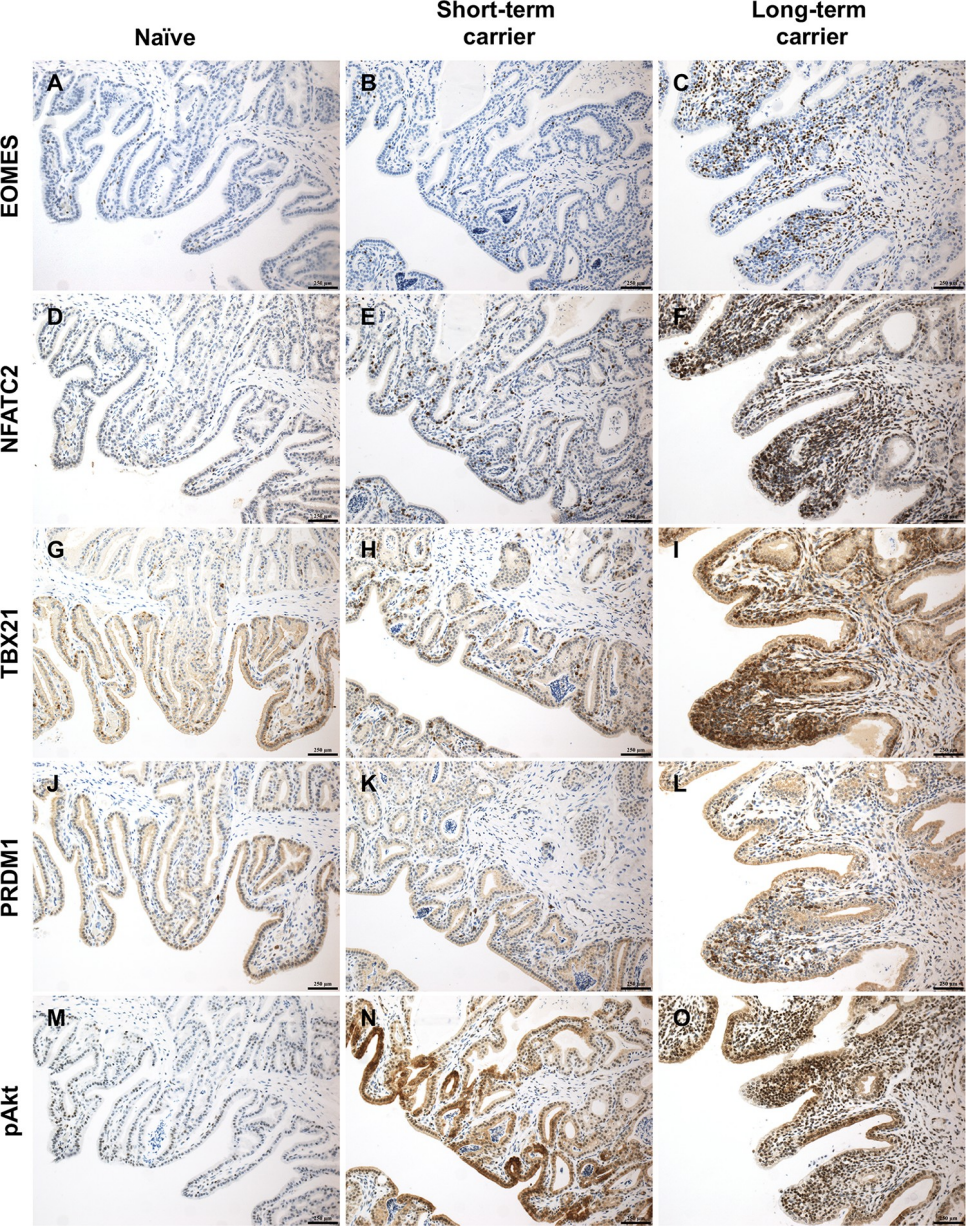
Analysis of a subset of transcription factors in the ampullae of naïve, short-term and long-term carrier stallions by immunohistochemistry determined the predominance of EOMES and NFATC2 in inflammatory infiltrates during EAV persistence. (A-C) EOMES-specific immunostaining. (D-F) NFATC2-specific immunostaining. (G-I) TBX21-specific immunostaining. (J-L) PRDM1-specific immunostaining. (M-O) Phosphorylated Akt-specific immunostaining. DAB. 200X. Bar = 250 μm.

**Fig 8:**
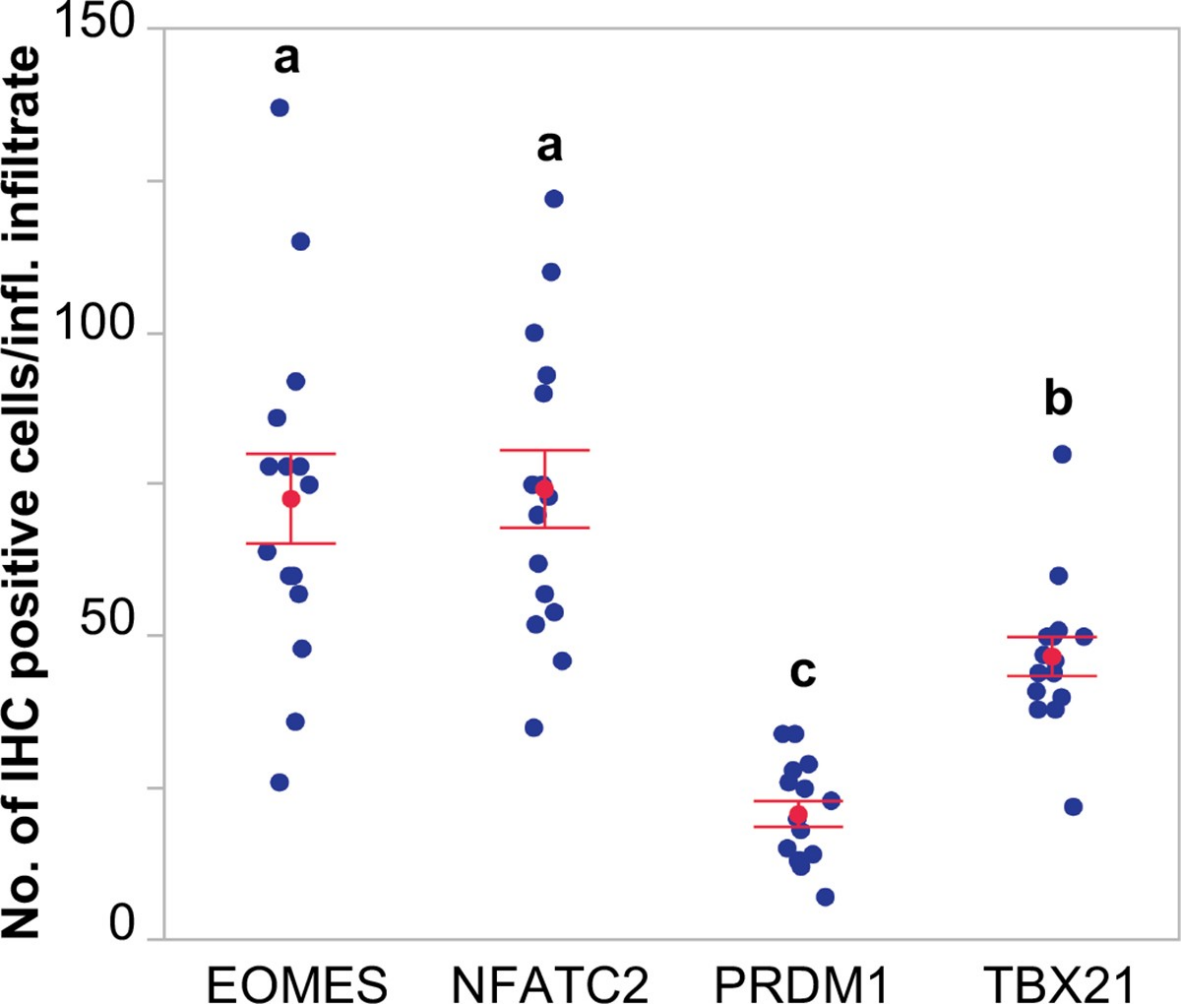
Quantitative immunohistochemical analysis of specific transcription factors (EOMES, NFATC2, PRDM1 and TBX21) in inflammatory infiltrates of long-term persistently infected stallions. The number of positive cells was determined in a total of five equivalent inflammatory infiltrates per stallion. Blue dots represent values for individual infiltrates, while the mean number of positive cells/inflammatory infiltrate ± standard error of the mean are represented in red, respectively. Letters (a, b and c) indicate statistically significant differences between transcription factors (*p*-values < 0.001).

To confirm our previous observation, we performed immunohistochemical staining for EOMES, TBX21, PRDM1 and NFATC2 in the ampullae of long-term, short-term carrier and naïve stallions. In agreement with our transcriptomic and RT-qPCR data, immunohistochemical (IHC) analysis demonstrated a significantly higher number of EOMES^+^, TBX21 (T-bet)^+^, PRDM1 (BLIMP-1)^+^ and NFATC2^+^ infiltrating lymphocytes in the ampullae of long-term carrier when compared to short-term carrier stallions (*p*-values < 0.05; Fig 7A–7L). Overall, the number of T lymphocytes expressing EOMES, TBX21 (T-bet) and NFATC2 was significantly higher (median score of 4 [>200 positive cells/five 40X magnification fields]), while those expressing PRDM1 (BLIMP-1) was moderate (median score of 3 [100–200 positive cells/five 40X magnification fields]). Subsequently, we compared the expression of EOMES, TBX21 (T-bet), NFATC2 and PRDM1 (BLIMP-1) in a total of five inflammatory infiltrates across long-term carrier stallions (n = 3) in order to determine their cellular predominance. This analysis revealed that a significantly higher number of T lymphocytes expressed EOMES and NFATC2, a moderate number expressed TBX21 (T-bet) and a lower proportion expressed PRDM1 (BLIMP-1; Fig 7A–7L and Fig 8), suggesting that EOMES and NFATC2 constitute the main drivers of the inflammatory response in long-term carrier stallions. Unfortunately, no cross-reactive antibodies against equine IRF1, IRF4, STAT1 and STAT2 could be identified and, therefore, we could not demonstrate the cellular expression of these TFs in inflammatory infiltrates.

**Fig 9:**
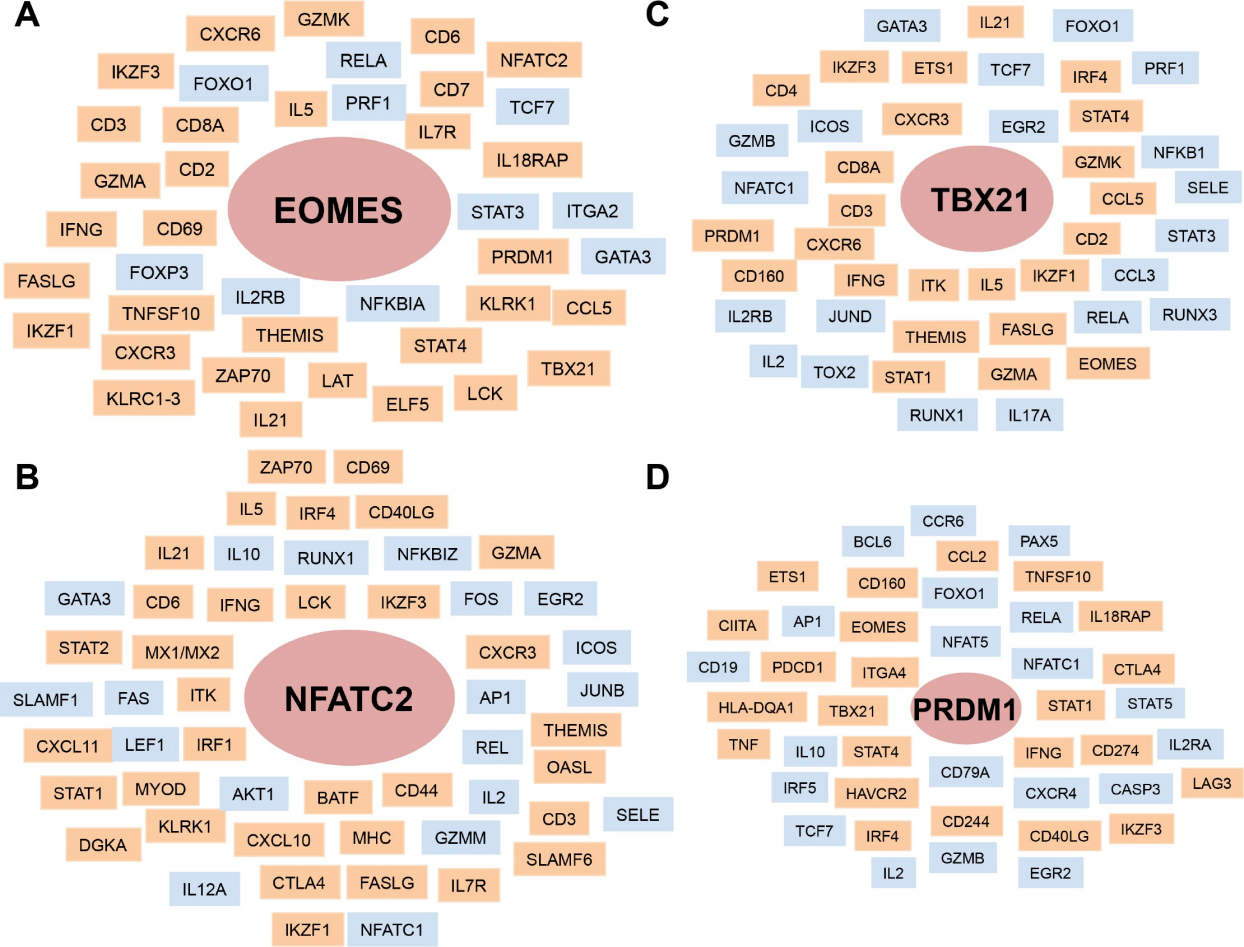
Putative genes regulated by or interacting with the transcription factors analyzed in the ampullae of long-term EAV carrier stallions. (A) *EOMES*. (B) *NFATC2*. (C) *TBX21*. (D) *PRDM1*. Gene lists were generated from the immune-database Immuno-Navigator, the Ingenuity Knowledgebase and literature search. Transcription factors of interest are shown in circles, where the size of the circle is indicative of their predominance in inflammatory infiltrates as determined in this study. DEGs identified during EAV persistence are depicted in orange while those that were not differentially expressed are shown in light blue.

To further assess the relationship of this subset of TFs with the DEGs observed in the ampullae of long-term carrier stallions, we identified specific targets and positively correlated genes under their transcriptional regulation based on a database search using the Ingenuity Knowledgebase (IPA) and Immuno-Navigator database [36]. Among their target/correlated genes, several of these were identified in the DEG dataset derived from the ampullae of long-term carrier stallions (Fig 9 and S3 Table). Taken together, we determined that EOMES and NFATC2 constitute the most predominant TFs in inflammatory infiltrates among those analyzed, with several DEGs under their transcriptional regulation at the site of persistence.

### EAV long-term persistence is associated with the upregulation of inhibitory receptors at the site of persistent infection

Since chronic, persistent viral infections have often been associated with the development of T-cell exhaustion and we identified a set of TFs that could also be associated with this process (namely EOMES, NFATC2, PRDM1 [BLIMP-1], IRF4 and BATF), we hypothesized that a similar immune process is likely to be involved in the reproductive tract during EAV long-term persistence. Therefore, we evaluated the gene expression profile of several inhibitory receptors previously identified to be associated with this T-cell exhaustion [37–39]. While whole transcriptome analysis identified an upregulation of *CD160* and *CD274* (programmed cell death 1 ligand 1 [PD1-L1]) along with the aforementioned TFs in long-term carrier stallions (Table 2), analysis of other receptors of interest (i.e. programmed cell death 1 [*PDCD1* or PD-1], *CTLA-4*, *CD244*, lymphocyte-activation gene 3 [*LAG3*], T-cell immunoglobulin and mucin-domain containing-3 [*HAVCR2* or TIM-3] and semaphorin 7A [*SEMA7A*]) was hampered by low coverage of these genes in the short-term carrier stallions. Therefore, we used RT-qPCR to detect the expression of these genes in the inflammatory infiltrates of long-term and short-term persistently infected stallions. Interestingly, a significant expression of *CD160*, *CD244*, *CD274*, *CTLA-4*, *HAVCR2*, *PDCD1* and *SEMA7A* mRNA was evident in both long-term (high expression, *p*-values ≤ 0.0045) and short-term carrier (intermediate expression, *p*-values ≤ 0.0334) compared to naïve stallions (Fig 5A). *LAG3* was uniquely upregulated in the ampullae of long-term carrier stallions (*p*-values < 0.0001), with no statistically significant differences between short-term carrier and naïve stallions (*p*-value = 0.2729). IHC analysis demonstrated a significantly higher number of CTLA-4^+^ T lymphocytes in inflammatory infiltrates of long-term carrier stallions, while these were very low in tissues derived from short-term carrier or naïve stallions (*p*-values < 0.05, Fig 4G–4I).

Finally, we analyzed the expression of two TFs (activator protein 1 [AP-1 or *JUNB*] and Fos proto-oncogene [*FOS* or AP-1 transcription factor subunit]), previously shown to be downregulated during T-cell exhaustion [40]. No significant differences were identified in the relative expression of *JUNB* between groups (*p*-values > 0.05) and, although expression of *FOS* was significantly higher in long-term (*p*-value = 0.0162) and short-term carrier (*p*-value = 0.0253) compared to naïve stallions, no differences were observed between the former two groups (*p*-value = 0.5895, Fig 5A). In summary, we have identified a strong upregulation of inhibitory receptor transcripts and a higher number of CTLA-4^+^ T lymphocytes in inflammatory infiltrates of long-term carrier stallions, while no alterations in the expression of AP-1 related TFs were observed.

### Long-term EAV persistence is associated with an upregulation of *CXCL16* and its receptor, *CXCR6*, in the stallion reproductive tract

**Fig 10:**
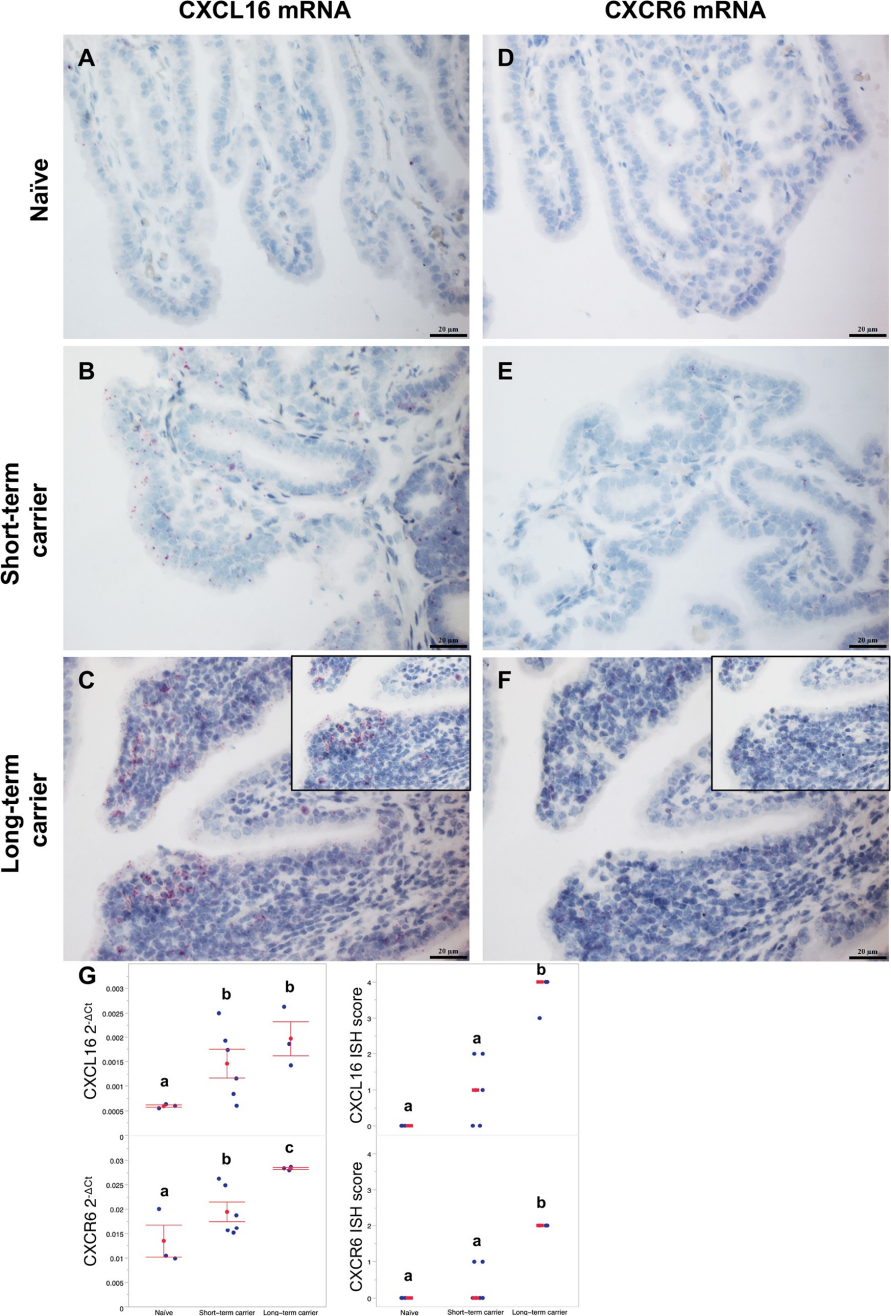
*CXCL16* and *CXCR6* expression in the ampullae of naïve (n = 3), EAV short-term (n = 6) and long-term carrier stallions. (A-C) *CXCL16* is significantly upregulated in the ampullae of EAV long-term persistently infected stallions (C) compared to short-term carrier (B) and naïve stallions (A) as determined by ISH. *CXCL16* is predominantly expressed in the mucosal epithelium and lymphocytic infiltrates (C, inset), especially in the luminal area. *CXCL16*-specific mRNA RNAscope ISH, Fast Red, 400X. Bar = 20 μm. (D-F) *CXCR6* is significantly upregulated in the ampullae of EAV long-term persistently infected stallions (F) compared to short-term carrier (E) and naïve stallions (D) as determined by ISH. *CXCR6* was expressed in lymphocytic infiltrates and co-localized with *CXCL16* expression in inflammatory cells (F, inset). *CXCR6*-specific mRNA RNAscope ISH, Fast Red, 400X. Bar = 20 μm. (G) Higher expression of *CXCL16* and *CXCR6* in the ampullae of long-term carrier stallions (n = 3) was quantitatively determined by (left to right) TaqMan RT-qPCR and RNAscope ISH. Blue dots represent values for individual stallions, while mean 2^-ΔCt^ values ± standard error of the mean and median scores are represented in red, respectively. Letters indicate statistically significant differences between groups (*p*-value<0.05).

Previously, we have demonstrated that the expression of CXCL16 is enhanced in the reproductive tract of long-term persistently infected stallions [30]. In agreement with this observation, whole transcriptome analysis of the ampullae identified the differential upregulation of *CXCL16* in this group compared to short-term carrier and naïve stallions (FDR ≤ 0.00005, Table 2). RT-qPCR and RNAscope *in situ* hybridization (ISH) were used to quantify and localize the expression of *CXCL16* in the ampullae. As previously reported, *CXCL16* expression was abundant in the glandular epithelium and inflammatory infiltrates of long-term carrier compared to short-term carrier stallions (median ISH score = 4, *p*-value = 0.0256, Fig 10A–10C and Fig 10G). Interestingly, the highest expression was observed in the luminal epithelium and inflammatory infiltrates adjacent to the lumen (Fig 10C). Similarly, we evaluated the expression of *CXCR6* at the site of persistence. Even though the expression of *CXCR6* mRNA was comparatively lower to that of *CXCL16* mRNA (Fig 10D–10G), RT-qPCR and RNAscope ISH analysis demonstrated an evident upregulation of *CXCR6* in long-term carrier stallions compared to the other groups (*p*-values ≤ 0.0093 and *p*-values < 0.05, respectively [Fig 10G]). While *CXCR6* expression in short-term carrier stallions was higher than in naïve stallions as determined by RT-qPCR (*p*-value = 0.0086), no significant differences were identified by RNAscope ISH (*p*-value > 0.05; Fig 10G). In contrast to *CXCL16*, *CXCR6* was solely expressed by lymphocytes present in inflammatory infiltrates and co-localized with *CXCL16* mRNA signal (Fig 10C and Fig 10F). Since CXCL16/CXCR6 axis is associated with the activation of the mammalian target of rapamycin/RAC-alpha serine/threonine protein kinase (mTOR/Akt) signaling pathway [41], we evaluated the expression of phosphorylated Akt (pAkt) in the ampullae of long-term carrier stallions. While its expression was high and variable (ranging from scattered to widespread) in glandular epithelial cells from both long-term and short-term carrier stallions, a significantly higher number of pAkt^+^ inflammatory cells were observed in the former group (*p*-value = 0.0094, Fig 7M–7O).

To determine the relationship of *CXCL16* and *CXCR6* in the context of the DEGs identified in long-term persistently infected stallions, we retrieved a list of genes positively correlated with both *CXCL16* and *CXCR6* (*r* ≥ 0.5) from the Immuno-Navigator database [36] and intersected it to the DEGs. Among 1,309 genes positively correlated with *CXCL16* (*r* ≥ 0.5) that were retrieved from the database, 59 were differentially expressed in long-term persistently infected stallions (Table 3). Toll-like receptor 8 (*TLR8*), integrin subunit alpha X (*ITGAX*, also known as CD11c), TYRO protein tyrosine kinase binding protein (*TYROBP*) and NOD-like receptor family CARD domain containing 4 protein (*NLRC4*) were among the strongly correlated genes (*r* ≥ 0.8; Table 3 and S4 Table). Similarly, among 192 genes positively correlated with *CXCR6* (*r* ≥ 0.5) that were retrieved from the database, 45 were differentially expressed in this group (Table 3 and S4 Table). Even though the correlation to *CXCR6* was moderate (*r* ≤ 0.75), several granzymes and effector molecules, TFs and T cell immune synapse components were identified. Interestingly, the expression of the chemokine receptor *CXCR3* was positively correlated to that of *CXCR6*. In summary, we demonstrated the upregulation of *CXCL16* and *CXCR6* at the site of persistence in long-term EAV carrier stallions, with expression of *CXCL16* in glandular epithelial cells and lymphocytes, whereas the expression of *CXCR6* was restricted to lymphocytic infiltrates. Intersection of genes positively correlated to CXCL16/CXCR6 axis obtained from public databases with our DEG dataset demonstrated that several of these genes were upregulated in the ampullae during long-term viral persistence and, therefore, directly or indirectly associated with the CXCL16/CXCR6 axis.

**Table 3:** Top 20 genes positively correlated to CXCL16 and CXCR6 and differentially expressed in long-term carrier stallions. Correlated genes were identified using the immune database Immuno-Navigator and intersected with the differentially expressed genes identified in the long-term carrier stallions (n = 3) compared with the short-term carrier stallions (n = 6).

Correlated to CXCL16			Correlated to CXCR6		
Gene symbol	Gene name	r	Gene symbol	Gene name	r
TLR8	Toll-like receptor 8	0.90	GZMK	Granzyme K	0.76
ITGAX	Integrin subunit alpha X (CD11c)	0.88	GZMA	Granzyme A	0.70
PTPRE	Protein tyrosine phosphatase, receptor E	0.84	EOMES	Eomesodermin	0.67
TYROBP	TYRO protein tyrosine kinase binding protein	0.84	CD7	CD7 molecule	0.66
			IL7R	Interleukin 7 receptor	0.66
SPI1	Transcription factor PU.1	0.84	CD2	CD2 molecule	0.66
CTSS	Cathepsin S	0.82	NELL2	Neural EGFL like 2	0.66
SAT1	Diamine acetyltransferase 1	0.82	THEMIS	Thymocyte selection associated	0.66
NLRC4	NOD-like receptor family CARD containing 4 protein	0.81	ITK	Interleukin 2 inducible T-cell kinase	0.65
			CD3E	CD3 epsilon chain	0.64
FCER1G	High affinity IgE receptor subunit gamma	0.81	CD8A	CD8 alpha chain	0.64
MPEG1	Macrophage-expressed gene 1	0.78	FASLG	Fas ligand	0.64
MYO1F	Myosin If	0.76	SYTL2	Synaptotagmin-like 2	0.64
CSF1R	Colony stimulating factor receptor 1	0.76	KLRK1	Killer cell lectin like receptor 1	0.63
CD68	CD68 molecule	0.75	CD3D	CD3 delta chain	0.63
FYB	FYN-binding protein 1	0.75	STAT4	Signal transducer and activator of transcription 4	0.62
AOAH	Acyloxyacyl hydrolase	0.74			
MNDA	Myeloid cell nuclear differentiation antigen	0.74	UBASH3A	Ubiquitin associated and SH3 domain containing A	0.62
LYZ	Lysozyme	0.73			
PLXNC1	Plexin C1	0.72	TRAT1	T-cell receptor associated transmembrane adaptor 1	0.62
C3AR1	Complement C3a receptor 1	0.72			
CTSB	Cathepsin B	0.71	SH2D1A	SH2 domain containing 1A	0.61
			CD6	CD6 molecule	0.60

### Host genetics shape the gene expression profile in the reproductive tract following EAV infection

**Fig 11:**
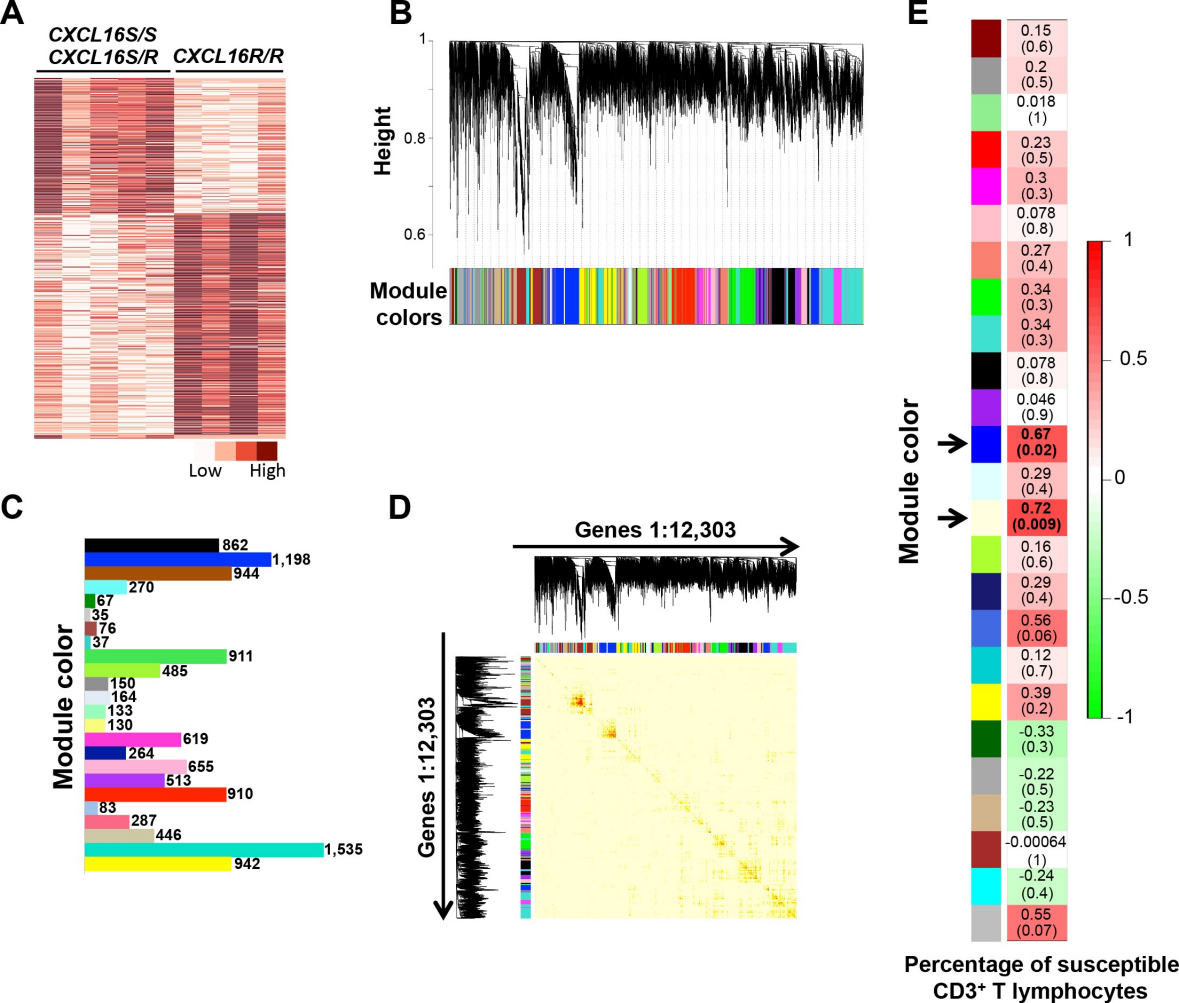
Differential gene expression and weighted gene co-expression network analysis in stallions homozygous and heterozygous for the *CXCL16S* allele (*CXCL16S/CXCL16S* and *CXCL16S/CXCL16R*; n = 5). (A) A total of 542 genes were differentially expressed between animals exhibiting different genotype (i.e. between stallions carrying the susceptibility allele [*CXCL16S*, n = 5] and those who did not [homozygous for *CXCL16R*, n = 4]) and following EAV infection. A lower proportion (188 genes) were upregulated in stallions carrying the *CXCL16S* allele. Darker reds are indicative of a higher expression. The interactive heatmap can be found herehttp://rpubs.com/pouyadini/379472. (B and C) Gene module identification as determined by weighted gene co-expression network analysis (WGCNA). A total of 24 gene modules with varying number of genes were identified among 12,303 selected genes. (D) Gene co-expression network construction for selected genes. The heatmap depicts the topological overlap (correlation between the expression profiles of each pair of genes). Each row and column correspond to a gene, where light color denotes low topological overlap with progressively darker color indicating higher topological overlap. Dark squares along the diagonal represent gene modules. (E) Module-trait correlation heatmap based on the correlation analysis of module eigengenes (MEs) and percentage of CD3^+^ T lymphocytes susceptible to *in vitro* EAV infection in stallions (n = 12). Pearson correlation values (*r*) are shown for each module along with the respective *p*-value within parenthesis. The *blue* and *lightyellow* modules (arrows) presented a significant positive correlation with CD3^+^ T lymphocyte susceptibility (*p*-values < 0.05).

Recently, it has been determined that long-term persistent infection is associated with the presence of the dominant *CXCL16S* allele (*CXCL16S/CXCL16S* or *CXCL16S/CXCL16R*). Among the EAV-infected stallions used in this study (n = 9), all long-term carrier stallions (n = 3) were either homozygous or heterozygous for the *CXCL16S* allele (Table 1). While the majority of the short-term carrier stallions were homozygous for the *CXCL16R* allele (4/6, Table 1), 2/6 were heterozygous for the *CXCL16S* allele. Even though the genotype-trait association is strongly correlated, some stallions carrying the *CXCL16S* allele can stop viral shedding earlier in the course of infection for reasons that remain to be determined. When performing differential gene expression analysis based on whole transcriptome analysis from the ampullae following EAV infection, we observed that differences in the gene expression pattern were likely linked to the genetic background of the animal (i.e., *CXCL16* genotype, Fig 3A and Fig 11A). Therefore, we performed differential gene expression analysis between stallions that were homozygous or heterozygous for the *CXCL16S* allele (n = 5) and those homozygous for the *CXCL16R* allele (n = 4). A total of 542 DEGs were identified between genotype groups (FDR < 0.1), among which 188 genes (34.7%) were upregulated (log_2_ fold-change > 1 over *CXCL16R/CXCL16R* group) and 272 genes (50.2%) were downregulated (log_2_ fold-change < -1 over *CXCL16R/CXCL16R* group) in the ampullae of stallions carrying the *CXCL16S* allele (Fig 11A). The remaining DEGs (n = 82) presented a fold-change between 1 and -1. Functional annotation analysis of upregulated genes in the *CXCL16S* group demonstrated their involvement in biological processes associated with the immune response, with molecular functions mostly related to binding, catalytic, receptor and chemokine activity (S3 Fig). As observed for long-term carrier stallions, the upregulated genes were mostly involved in immune-related pathways, specifically related to T lymphocyte and NK cell responses. Conversely, downregulated genes in the *CXCL16S* group were associated with cell adhesion and extracellular matrix organization with relevant pathways related to integrin signaling, focal adhesion/cadherin signaling, extracellular matrix-receptor interaction and cytoskeletal regulation. Taken together, DGE analysis between *CXCL16S* and *CXCL16R* stallions demonstrated that the gene expression profile in the ampullae is driven by the host genotype, with a clear majority of upregulated genes associated with immune response functions.

### Weighted gene co-expression network analysis (WGCNA) reveals gene modules positively correlated with the CD3^+^ T lymphocyte susceptibility phenotype and *CXCL16* genotype

Co-expression network analysis allows the identification of groups of coordinately expressed genes (modules), which may represent specific transcriptional networks. Network analysis is therefore based on correlation analysis, whereby highly correlated genes often share biological features and these gene modules can be considered as “gene circuits” responsible for specific cellular functions [42–44]. Furthermore, network analysis allows the identification of highly connected “hub” genes within modules that likely represent control points. Since differential gene expression analysis may not explain all transcriptional interactions, we performed WGCNA in order to identify gene co-expression patterns associated with the *CXCL16* genotype and the CD3^+^ T lymphocyte susceptibility phenotype. A set of 12,303 genes were analyzed using WGCNA package in R (Department of Human Genetics and Department of Bioinformatics, University of California Los Angeles, Los Angeles, CA) [45, 46] and the percentage of susceptible CD3^+^ T lymphocytes to *in vitro* EAV infection was used as the quantitative trait (Table 1) [28, 31]. Network construction and module detection elicited a total of 24 modules (color-coded, Fig 11B–11D) with a range of 35 to 1,535 genes. Module eigengenes (ME, defined as the first principal component of a given module) were computed for each module and considered as a representative of the gene expression profiles in a specific module. Based on their module eigengenes, several modules showed a positive correlation with other modules in the network and clustered together in the eigengene dendrogram (S4 Fig).

To identify gene modules correlated with the CD3^+^ T lymphocyte susceptibility phenotype and *CXCL16* genotype, we tested the correlation between the ME and the trait (presence of CD3^+^ T cell susceptibility to EAV infection as demonstrated by flow cytometric analysis) [28, 31]. Two gene modules (namely *blue* [n = 1,198 genes] and *lightyellow* [n = 130 genes]) were positively correlated to the trait (*r* > 0.5, *p*-value < 0.05; Fig 11E) and clustered together in the eigengene dendrogram (S4 Fig). GO analysis (biological process) for the gene list derived from the *blue* module (S5 Table) demonstrated that these genes were involved in biological processes associated with the immune response. Interestingly, this module gathered several of the TFs shown to be associated with long-term EAV persistence including *IRF1*, *IRF4*, *BATF*, *EOMES*, *PRDM1*, *STAT1* and *STAT2*, among others. While no statistically significant GO terms were retrieved for the genes in the *lightyellow* module, it also contained immune-related genes among which *TBX21* and *NFATC2* were observed (S5 Table).

**Fig 12:**
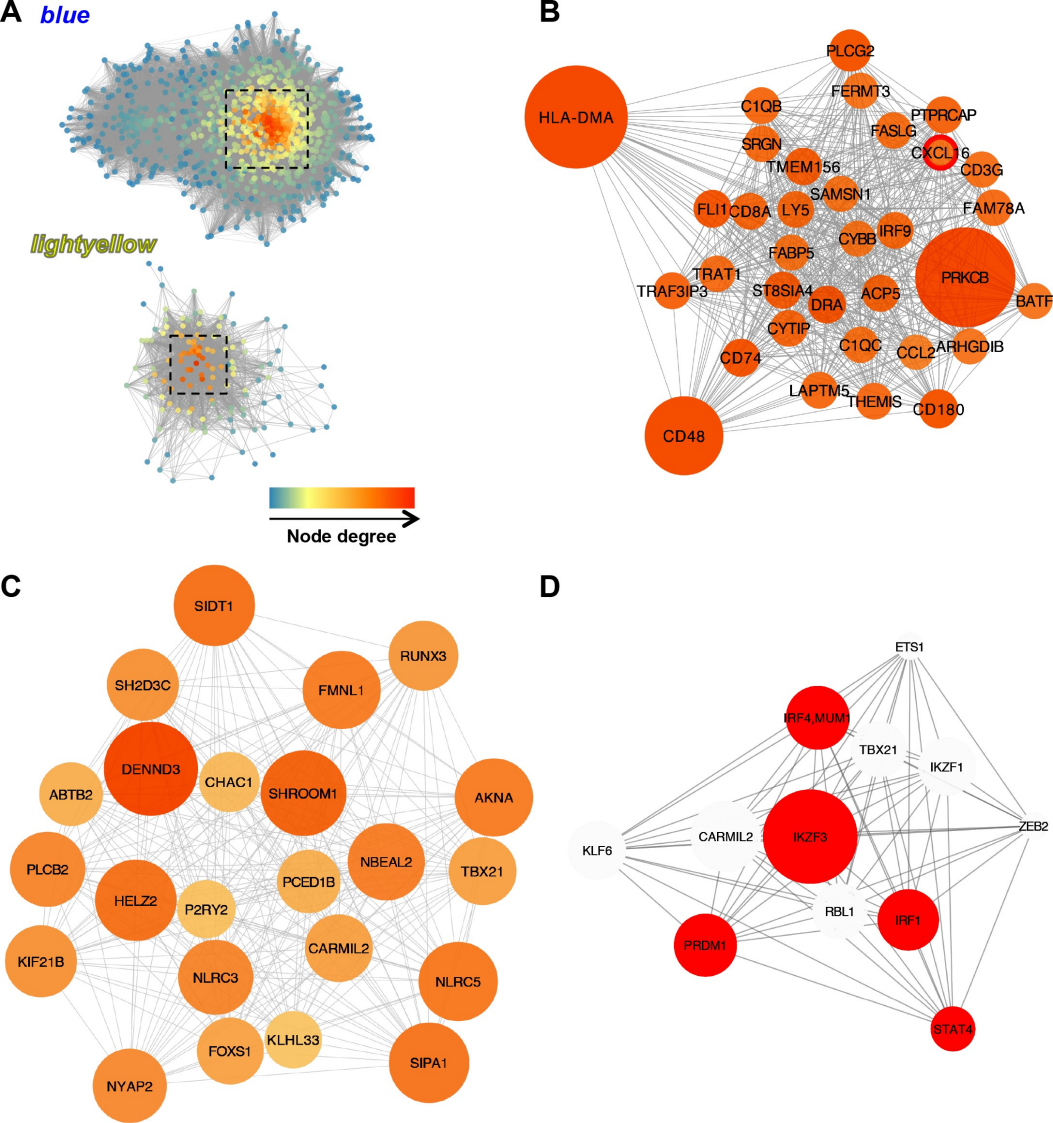
“Hub” gene and neighbor identification in the *blue* and *lightyellow* modules. (A) Network visualization of the *blue and lightyellow* modules (n = 1,195 genes and n = 130 genes, respectively). Highly connected, putative “hub” genes within the module are shown in the squared area. “Hub” genes were selected based on a module membership (MM) value ≥ 0.90, a *p*-value < 0.05 and gene significance (GS) ≥ 0.5. Node color reflects node degree (number of connections to other genes in the network), where darker reds indicate a higher degree. Network analysis was performed using the NetworkAnalyzer tool in Cytoscape. (B) CXCL16 first neighbors as determined by network analysis of the *blue* module. CXCL16 was identified as a “hub” gene within the *blue* module and several of its neighbor genes are involved in immune-related pathways such as *CCL2*, *BATF*, *CD3G*, *FASLG*, *IRF9*, *CD8A*, *THEMIS*, among others. Node color and size have been mapped to reflect node degree, where darker reds and larger nodes indicate a higher degree. (C) “Hub” genes in the *lightyellow* module network. (D) “Hub” transcription factor genes belonging the *green* module derived from the transcription factor network constructed from stallions carrying the *CXCL16S* allele (homozygous and heterozygous). Node size reflects node degree (number of connections to other genes in the network). Transcription factors identified as differentially expressed between stallions homozygous or heterozygous for *CXCL16S* and those homozygous for *CXCL16R* are colored in red. Interestingly, transcription factors that were over-represented during long-term EAV persistence are among the “hub” transcription factors identified in stallions homozygous or heterozygous for *CXCL16S*.

In order to identify the gene members that likely drive module expression, we selected highly connected “hub” genes within these modules. For this purpose, we set cut-off values for module membership values (MM ≥ 0.90), significance (*p*-value < 0.05) and gene significance (GS ≥ 0.5) (S6 Table) [47, 48]. MM values are computed for each gene as the correlation of its expression profile with the module eigengene of a given module and are highly related to the intramodular connectivity [45]. Since highly connected intramodular “hub” genes tend to have a high MM within their module, this measure can be used to identify “hub” genes [45]. Gene significance is the correlation of each gene’s expression profile to the trait and, thus, can be used as a measure of the biological relevance of a gene with respect to the trait of interest. Interestingly, among the top 20 “hub” genes (MM ≥ 0.95) in the *blue* module, we identified *CXCL16* and several other immune-related genes (Table 4 and S6 Table), which were also among *CXCL16* first neighbors (Fig 12A and 12B). The *lightyellow* module contained other important immune “hub” genes including AT-hook transcription factor (*AKNA*), Runt-related transcription factor 3 (*RUNX3*), *TBX21*, *NLRC3* and *NLRC5*, among others (Table 4, Fig 12A and Fig 12C and S6 Table).

**Table 4:** Top 20 “hub” genes from *blue* and *lightyellow* modules, both positively correlated with CD3^+^ T lymphocyte susceptibility phenotype and CXCL16 genotype. “Hub” genes selected based on MM≥0.90, *p*-value<0.05 and GS≥0.5.

*blue* Module Genes			*lightyellow* Module Genes		
Symbol	Name	MM	Symbol	Name	MM
PRKCB	Protein kinase C beta type	0.99	DENND3	DENN domain-containing protein 3	0.97
CD74	CD74 molecule	0.98	SHROOM1	Shroom family member 1	0.96
CD48	CD48 molecule	0.98	NBEAL2	Neurobeachin-like 2	0.96
CD180	CD180 molecule	0.98	HELZ2	Helicase with zinc finger 2	0.94
HLA-DMA	HLA class II histocompatibility antigen, DM alpha chain	0.98	NLRC5	NOD-like receptor family CARD containing 5 protein	0.93
ST8SIA4	ST8 alpha-N-acetyl-neuraminide alpha-2,8-sialyltransferase	0.97	SIDT1	SID1 transmembrane family member 1	0.93
TMEM156	Transmembrane protein 156	0.97	KIF21B	Kinesin family member 21B	0.93
PLCG2	Phospholipase C gamma 2	0.97	FMNL1	Formin-like protein 1	0.93
ACP5	Acid phosphatase 5	0.97	AKNA	AT-hook transcription factor	0.93
DRA	HLA class II histocompatibility antigen, DR alpha chain	0.97	NLRC3	NOD-like receptor family CARD containing 3 protein	0.93
CYTIP	Cytohesin 1 interacting protein	0.97	SIPA1	Signal-induced proliferation-associated 1	0.93
TRAT1	T-cell receptor-associated transmembrane adapter 1	0.96	FOXS1	Forkhead box S1	0.93
LAPTM5	Lysosomal protein transmembrane 5	0.96	PCED1B	PC-esterase domain-containing 1B	0.92
C1QB	Complement C1q B chain	0.96	CARMIL2	Capping protein regulator and myosin 1 linker 2	0.92
FAM78A	FAM78A family with sequence similarity 78 member A	0.96	SH2D3C	SH2 domain containing 3C	0.91
TRAF3IP3	TRAF3 interacting protein 3	0.96	ABTB2	Ankyrin repeat and BTB domain containing 2	0.91
FLI1	Fli-1 proto-oncogene	0.96	KLHL33	Kelch-like protein 33	0.91
CXCL16	C-X-C motif chemokine ligand 16	0.96	TBX21	T-box transcription factor TBX21	0.91
IRF9	Interferon regulatory factor 9	0.96	NYAP2	Neuronal tyrosine-phosphorylated phosphoinositide-3-kinase adaptor 2	0.91
SRGN	Serglycin	0.96	CHAC1	ChaC glutathione specific gamma-glutamylcyclotransferase 1	0.90

Finally, since TFs are intricately associated with the control of gene expression networks, we performed a weighted-gene co-expression analysis specifically on 494 TFs derived from *CXCL16S* stallions. Network construction and module detection elicited a total of five modules with a range of 51 to 188 genes. From the module eigengene dendrogram, we clearly identified a module (*green*, 51 genes [S7 Table and S4 Fig]) with a different co-expression pattern and which contained several TFs of interest involved in the T lymphocyte-mediated response including *EOMES*, *IRF1*, *IRF4*, *TBX21*, *PRDM1* and *NFATC2* (S7 Table). “Hub” TFs in the *green* module (MM ≥ 0.90 and *p*-value < 0.05,) can be depicted in Fig 12D. Interestingly, 5/12 TFs were differentially expressed between *CXCL16S* and *CXCL16R* (homozygous) stallions and presented a high degree of connectivity (Fig 12D). In summary, we identified two specific gene modules positively correlated with the CD3^+^ T lymphocyte susceptibility phenotype and the *CXCL16* genotype. We determined that *CXCL16* is clearly a “hub” gene and highly interconnected within a transcriptional module encompassing diverse immune-related genes including TFs associated with EAV persistence.

## Discussion

**Fig 13:**
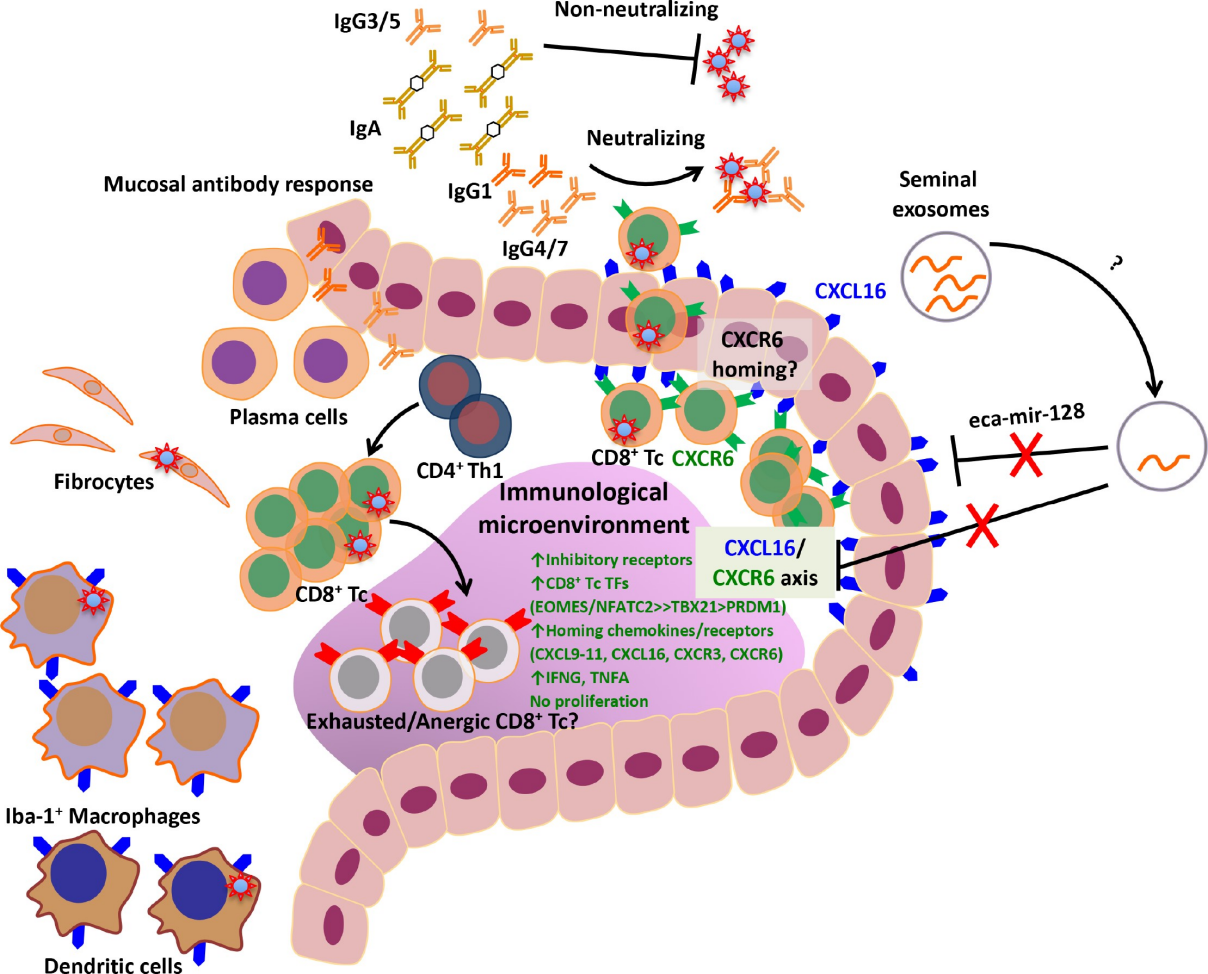
Schematic representation of the current knowledge of EAV persistence in the reproductive tract of the stallion. EAV tropism during persistent infection is associated with CD8^+^ T lymphocytes and CD21^+^ B lymphocytes infiltrating the ampullae, fibrocytes, tissue macrophages and dendritic cells. Long-term persistence is associated with a strong inflammatory response primarily mediated by CD8^+^ T lymphocytes and a smaller population of CD4^+^ T helper 1 lymphocytes. Additionally, local plasma cells produce diverse EAV-specific immunoglobulin isotypes with varying neutralizing abilities that are shed into the seminal plasma. Long-term persistence is associated with an enhanced expression of the chemokine CXCL16 in the mucosal epithelium and lymphocytes and with the downregulation of seminal exosome eca-mir-128, a putative modulator of the CXCL16/CXCR6 axis. Expression of the CXCL16 receptor, CXCR6, is also upregulated in lymphocytes, and along with other chemokines and receptors (CXCL9-11, CXCR3) likely mediates specific homing of infected lymphocytes into the reproductive tract of the stallion and migration of these cells across the epithelial lining, and drives an immunologically unique microenvironment that favors viral persistence. The inflammatory process is accompanied by upregulation of inhibitory receptors (PDCD1, CTLA-4, among others) and predominance of the transcription factors EOMES and NFATC2, potential mediators of CD8^+^ T lymphocyte hyporesponsiveness that leads to viral persistence. CD8^+^ Tc, CD8^+^ T lymphocytes; CD4^+^ Th1, CD4^+^ T-helper 1 lymphocytes; CD21^+^ Bc, CD21^+^ B lymphocytes.

The mechanisms by which EAV persists in the male reproductive tract are not fully understood and have been recently the subject of extensive investigation in our laboratory [23–25, 28–30]. It has been demonstrated that long-term persistence is associated with the presence of a specific allele encoding the chemokine CXCL16 (namely, *CXCL16S*), with a dominant pattern of inheritance and with EAV receptor activity [26, 28, 29]. Additionally, EAV has the ability to persist despite the induction of a strong systemic immune response and local inflammatory and mucosal antibody responses at the site of persistence [23, 24]. Recent studies showed that long-term persistence is associated with an upregulation of CXCL16 at the site of persistence along with a downregulation of the microRNA eca-mir-128 in seminal exosomes, a putative modulator of *CXCL16* [30]. These provide a strong premise that EAV employs a complex strategy to evade host immunity and that host factors play a critical role in long-term viral persistence. However, the immunopathogenesis of viral persistence in the reproductive tract remains to be elucidated (Fig 13). We hypothesized that long-term EAV persistence induces a specific immunological microenvironment in the stallion reproductive tract that facilitates evasion of host immunity. The study presented herein is the first one undertaken to evaluate transcriptional changes following EAV infection, explicitly providing insight into the molecular elements driving the local host response during long-term viral persistence in the stallion reproductive tract.

Global transcriptome analysis of the ampullae derived from long-term carrier stallions along with inflammatory cell immunophenotyping revealed a T lymphocyte-mediated response predominantly driven by a CD8^+^ T lymphocyte infiltration and functionally evidenced by the upregulation of CD8^+^ T lymphocyte-specific transcripts including effector molecules (e.g. granzymes and Fas ligand), chemokines/cytokines (e.g. *CCL2*, *CCL5*, *IFNG*, *TNFA*) and TFs (e.g. *BATF*, *EOMES*, *NFATC2*, *TBX21*, *IRF1*, *IRF4*, *STATs*). Additionally, there is transcriptional evidence that support the involvement of infiltrating CD4^+^ T lymphocytes in the Th1 pathway. While a higher number of DEGs were observed between long-term carrier and naïve stallions, there was an overlap of common DEGs in long-term and short-term carrier stallions. These overlapping (upregulated) genes were mostly associated with adhesion properties and leukocyte migration and, therefore, likely involved in both the active inflammatory response and in its resolution in the long-term carrier and those stallions that cleared EAV infection, respectively. Both experimentally and naturally infected long-term carrier stallions used in this study were infected with the same strain of EAV (EAV KY84) and no differences were observed in regard to neutralizing antibody levels in serum and semen, viral output in semen, number of EAV-infected cells and viral tropism within the reproductive tract, inflammatory response, expression levels of seminal exosome-associated miRNA eca-mir-128, and the gene expression profile in the ampullae among these stallions [23, 30]. A recent study on EAV intra-host evolution also demonstrated that EAV KY84 evolutionary rate and genomic sites under selective pressure in the reproductive tract are similar between these experimentally and naturally infected stallions [49].

Interestingly, immunophenotypic characterization of the inflammatory infiltrates in long-term carrier stallions confirmed the scattered inflammatory cells expressing AR. EAV persistence is testosterone-dependent [8, 22] and the immunomodulatory effects of androgens in virus-specific adaptive immune responses have been extensively studied with other viruses [50–52]. The expression of AR in the inflammatory infiltrates clearly indicates that androgens can have a direct impact in the modulation of local effector functions and, thus, in the maintenance of persistently infected cells. However, further studies are required to elucidate the role of AR-responsive elements and testosterone during persistent infection.

Chronic viral infections can progressively trigger CD8^+^ T lymphocyte hyporesponsiveness (T-cell exhaustion, T-lymphocyte dysfunction or anergy) [38, 39, 53]. This is related to the hierarchical loss of CD8^+^ T lymphocyte functional properties including proliferation, cytokine production (e.g., IFN-γ and IL-2) and cytolytic responses [38, 39]. Although the molecular signatures of T-cell exhaustion are not fully comprehended [38–40, 42, 53–55], the overexpression of cell surface inhibitory receptors (e.g., PD-1, CTLA-4 and others) mainly mediates CD8^+^ T lymphocyte dysfunction [38, 39, 56]. Here, we demonstrated the upregulation of inhibitory receptor transcripts including *PDCD1* and its ligand, PD-L1 (*CD274*) and TFs (*EOMES*, *NFATC2* and *PRDM1*) that may be involved in the inability of local CD8^+^ T lymphocytes to clear EAV infection. The similar expression levels of *IL2* between long-term and short-term carrier stallions and lack of Ki67 expression are indicative of the limited proliferative capacity of local CD8^+^ T lymphocytes. Surprisingly, we observed an upregulation of *IFNG* and *TNFA* transcripts and granzymes during long-term persistence. Previous studies have reported a similar trend despite the poor *ex vivo* cytotoxic activity of CD8^+^ T lymphocytes and suggested that alterations in vesicle trafficking and/or cytoskeletal reorganization could be responsible for the poor cytotoxic activity [40]. Even though GO enrichment analysis on downregulated genes from long-term carrier stallions did not retrieve statistically significant biological processes, these genes are likely involved in mesenchyme migration, cell-substrate junction assembly and actin filament organization. Therefore, additional studies to understand the relationship between vesicle transport/cytoskeletal reorganization and CD8^+^ T lymphocyte functionality during EAV persistence are required. In order to fully comprehend the functional status of local CD8^+^ T lymphocytes during persistence, future studies will be focused on immunophenotyping purified CD8^+^ T lymphocytes derived from the ampullae via multicolor flow cytometry and single-cell RNAseq combined with analysis of epigenetic modifications and functional assays (i.e. cytotoxicity and cytokine production) to assess local CD8^+^ T lymphocyte functionality *ex vivo*.

In this study, we identified the predominance of the CD8^+^ T lymphocyte-associated TFs EOMES and NFATC2 during long-term persistence. EOMES and TBX21 (T-bet), two T-box transcription factors, are key drivers of CD8^+^ T lymphocyte differentiation and their balance is essential for functional differentiation [57]. Interestingly, it has been demonstrated that CD8^+^ T-bet^dim^ EOMES^high^ T lymphocytes express high levels of surface inhibitory receptors with poor effector functions during chronic human immunodeficiency virus infection [58]. Along with other members of the NFAT family, NFATC2 plays a versatile role during both T cell activation and T cell hyporesponsiveness in its monomeric/homodimeric form as well as by formation of NFAT::AP-1 and NFAT::FOXP3 TF complexes [54]. While the cooperation of NFAT/AP-1 leads to a functional immune response, its imbalance can induce anergy or exhaustion and the lack of cooperation with AP-1 strongly drives expression of exhaustion-related inhibitory receptor genes [54, 59]. Also, NFATC2 cooperates with IRF4 at key genomic loci that control the transcriptional signature of exhausted T cells [55]. Consequently, it is reasonable to hypothesize that these two overabundant TFs may govern CD8^+^ T lymphocyte hyporesponsiveness during long-term EAV persistence in the reproductive tract. Further studies to identify their direct targets in the reproductive tract via chromatin immunoprecipitation sequencing (ChIP-seq) and elucidate their functional relationship using siRNA-mediated gene silencing on purified CD8^+^ T lymphocytes derived from the ampulla of long-term persistently infected stallions are warranted.

It has been previously suggested that EAV-infected lymphocytes present a restricted homing pattern allowing them to persist within the male reproductive tract [23]. Here, we identified a group of C-X-C motif chemokines and receptors associated with T lymphocyte homing. Specifically, *CXCL9*, *CXCL10* and *CXCL11*, and their receptor, *CXCR3*, may likely be associated with specific lymphocyte homing into reproductive tract tissues as observed following *Chlamydia trachomatis* infection [60]. However, the predominance of *CXCL16* and its receptor, *CXCR6*, is a distinctive feature of long-term EAV persistence and likely involved in recruitment of CD8^+^ T lymphocytes and other inflammatory cells into the site of persistence [61–64]. The expression of CXCL16 in the glandular epithelium and lymphocytic infiltrates along with its co-localization with CXCR6^+^ lymphocytes closely associated with the glandular epithelium emphasize their likely functional relationship. Given the presence of intra- and sub-epithelial lymphocytes and the close association of infected lymphocytes with the glandular epithelium [23], it is likely that the CXCL16/CXCR6 chemokine axis drives the chemotaxis of infected lymphocytes through the epithelial lining and contributes to the process of viral shedding in semen. Additional *in vitro* studies evaluating the role of the CXCL16/CXCR6 chemokine axis in chemotaxis of purified CD8^+^ T lymphocytes derived from the ampulla of persistently infected stallions will involve the use of recombinant CXCL16S and CXCL16R isoforms as well as anti-CXCR6 and pertussis toxin as a Gi-protein coupled receptor inhibitor for CXCR6. Even though the mucosal epithelium expresses high levels of CXCL16, recent studies demonstrated that virus is not harbored in the epithelial cells of the ampullae or other accessory sex glands [23]. This observation is supportive of the fact that ongoing studies in our laboratory suggest that EAV requires additional cellular entry factors, among which vimentin seems to play an important role. Thus, the lack of vimentin expression in the mucosal epithelium may be determinant for the lack of susceptibility of CXCL16^+^ epithelial cells in the ampullae to EAV infection [23]. While initial experiments involved vimentin knock-out and overexpression of CXCL16 in HEK293 cells, additional *in vitro* experiments using equine-derived cell lines (equine endothelial cells and ampulla-derived fibroblasts) are in progress.

Recent studies demonstrated the strong association between the *CXCL16* genotype and the establishment of long-term EAV persistence [28, 29]. Herein, we showed that the host’s genotype also drives the gene expression profile in the ampulla following EAV infection. While EAV did not establish long-term persistence in 2/5 *CXCL16S* stallions following experimental infection, their gene expression profile had a similar trend to that observed in long-term persistently infected, *CXCL16S* stallions. Since the correlation is not complete [29], other factors such as breed, differences in immune responses, epigenetic modifications, among others may be responsible for clearance of EAV infection and are under investigation. Among these, we have previously demonstrated that the *CXCL16S* variant of *CXCL16* and the CD3^+^ T lymphocyte susceptibility phenotype associated with this allelic variant occur across horse breeds, although differences in penetrance between different breeds were observed [27, 29]. These differences also concur with the varying seroprevalence of EAV between breeds and imply that this trait has most likely appeared in a common ancestor to current horse breeds [27]. This suggests that mutations in *CXCL16* (*CXCL16S*) have permitted EAV adaptation to the horse population and, despite differences in penetrance, it has promoted the maintenance of EAV across horse breeds worldwide. Here, we demonstrated that the *CXCL16* genotype shapes the transcriptome profile in the ampullae; however, the use of mixed breed stallions precludes analysis of breed-specific differences. Further studies comparing the gene expression profiles between groups of EAV-infected stallions belonging to breeds with high and low allelic frequencies for *CXCL16S* are required to understand breed influence in the transcriptome.

The identification of genes correlated with the CXCL16/CXCR6 axis in our transcriptome dataset and abundance of CXCL16^+^ and CXCR6^+^ cells determined that this axis is significantly involved in shaping the local inflammatory response. Network analysis detected two important modules of co-expressed genes significantly correlated with the CD3^+^ T lymphocyte susceptibility trait and, thus, with the CXCL16 genotype. This analysis identified that CXCL16 is a “hub” gene, providing strong evidence that this chemokine plays a critical role in the local CD8^+^ T lymphocyte response along with other co-expressed gene neighbors that are involved in this specific gene module. This module included several immune-related genes associated with long-term EAV persistence, suggesting its significant implication in the regulation of the local immune response. Further studies are required to fully comprehend the gene interactions within this specific module and the functional role of CXCL16. Similarly, we defined a TF network in *CXCL16S* stallions and identified that most of the TFs identified by TF site enrichment and quantitative IHC analyses were related members of a single TF module. Taken together, these findings indicate that there are specific transcriptional modules strongly associated with *CXCL16* and a network of closely related TFs that cooperatively drive the gene expression profile during EAV persistence. However, additional studies to understand their functional relationship as well as their role in the modulation of the CXCL16/CXCR6 axis in the reproductive tract are warranted.

Whether EAV persistence is the result of the specific immunological environment in the reproductive tract (i.e., ampullae) or EAV infection leads to the conditioning of a specific immune milieu in the reproductive tract of persistently infected stallions that favors its long-term maintenance is still to be determined. However, this as well as previous studies from our laboratory strongly support the latter, suggesting that persistent EAV infection shapes multiple aspects of the immunological microenvironment within the reproductive tract (i.e. the CD8^+^ T lymphocyte response and the chemokine/chemokine receptor profile) that favors the maintenance of long-term persistence in stallions with *CXCL16S* genotype. Further sequential (time-course) *in vivo* studies evaluating the process of establishment of EAV persistence are warranted in order to completely understand the immunopathogenesis of persistent infection in the stallion reproductive tract and the role of testosterone during this process.

In conclusion, the study presented herein identified that the local CD8^+^ T lymphocyte response during EAV persistent infection is orchestrated by specific TFs (mainly EOMES and NFATC2) and likely modulated by the upregulation of inhibitory receptors. Most importantly, this study provides further evidence of the implication of CXCL16 and its receptor, CXCR6, in the pathogenesis of persistent EAV infection and a linkage between the *CXCL16* genotype and the gene expression profile driven at the site of persistence (Fig 13). These findings strongly suggest that EAV exploits the CXCL16/CXCR6 chemokine axis in order to modulate local immune responses at the site of persistent infection. However, this response is complex and likely involves not only this chemokine axis but also CD8^+^ T lymphocyte hyporesponsiveness associated with either anergy or T-cell exhaustion. It is yet to uncover how these complex host factors, including the recently identified seminal exosome eca-mir-128, are functionally involved in the modulation of viral persistence in the stallion reproductive tract.

## Materials and methods

### Ethics statement

This study was performed in strict accordance with the recommendations in the Guide for the Care and Use of Laboratory Animals of the National Institutes of Health. The Institutional Animal Care and Use Committee (IACUC) at the University of Kentucky, Lexington, KY approved this protocol (number 2011–0888). Stallions were humanely euthanized by pentobarbital overdose following the American Veterinary Medical Association (AVMA) guidelines for the euthanasia of animals, and all efforts were made to minimize suffering.

### Cells and viruses

Peripheral blood mononuclear cells (PBMCs; University of Kentucky, Lexington, KY) were cultured in complete RPMI medium as previously described [31]. The virulent Bucyrus strain (VBS) of EAV [65] was used for *in vitro* infection of PBMCs for CD3^+^ T lymphocyte phenotyping while the KY84 strain of EAV (passage 1 in equine endothelial cells [EECs], University of Kentucky, Lexington, KY) [16] was used for experimental infection of stallions as previously described [23, 31].

### Stallions

A total of 12 adult (age ranging between 4–20 years old), sexually mature stallions were obtained from an outside vendor and used in the study, including naïve (n = 3), EAV experimentally (n = 8) and naturally (n = 1) infected (Table 1) [23, 24]. These were maintained at the UK Maine Chance Farm, University of Kentucky, Lexington, KY and confirmed seronegative (titer <1:4) for antibodies to EAV before initiation of the study according to the World Organisation for Animal Health (OIE) standardized virus neutralization test as previously described [18, 23]. Following experimental infection and monitoring for 726 days postinfection (see below), 2/8 stallions were classified as long-term persistently infected (long-term carriers, duration of viral shedding in semen >1 year) while 6/8 stallions were classified as short-term carriers (duration of viral shedding in semen approximately ≤1 year). In addition, a long-term, naturally infected carrier stallion was included in the study. A control group of naïve stallions (n = 3) remained unexposed and unvaccinated against EAV.

### Experimental infection and clinical sample collection

Eight stallions (L136 –L143) were intranasally inoculated in October 2011 with 3.75 x 10^5^ plaque-forming units per ml [PFU/ml] of tissue culture fluid containing the KY84 strain of EAV (passage 1 in EECs, University of Kentucky, Lexington, KY) [16] and delivered in 5 ml EMEM using a fenestrated catheter and monitored as previously described [12, 18, 23]. Clinical sample collection including semen samples to monitor viral persistence was performed as previously described [13, 18, 23, 24].

### Postmortem examination and tissue collection

Stallions were humanely euthanized by pentobarbital overdose following the American Veterinary Medical Association (AVMA) guidelines for the euthanasia of animals. Necropsy examination and tissue collection was performed two years post-infection (726 dpi). For this study, the ampullae was collected and used for downstream experiments. In order to collect comparable sections across individual stallions, the length of the ampulla was determined and 1 cm section at its midpoint was bilaterally and aseptically collected from each stallion during postmortem examination and archived at -80°C, snap-frozen in O.C.T. compound (Tissue-Tek, Sakura Finetek U.S.A., Torrance, CA) and stored at -80°C, or formalin-fixed and paraffin-embedded (FFPE) as previously described [23]. These tissues were subsequently used for all experimental procedures.

### Histopathology

Sections of formalin-fixed paraffin-embedded (FFPE) tissues (5 μm) were stained with hematoxylin and eosin (H&E) following a standard laboratory procedure prior to histological evaluation. Tissue sections were scrutinized by an experienced veterinary pathologist who was blinded as to the carrier status of the stallions, and a morphological diagnosis was provided as previously described [23].

### Antibodies

A panel of monoclonal and polyclonal antibodies against several cellular markers and transcription factors were utilized for immunohistochemical (IHC) staining (Table 5). IHC staining was performed using the Bond Polymer Refine Detection kit or the Bond Polymer Refine Red Detection kit (Leica Biosystems, Buffalo Grove, IL) as described below. A goat anti-mouse IgG conjugated with Alexa Fluor 488 (Life Technologies, Grand Island, NY) was used for immunofluorescence. Dual immunofluorescence staining for flow cytometric analysis was performed with mouse monoclonal anti-equine CD3 (clone UC F6G, University of California Davis, Davis, CA) and anti-EAV non-structural protein 1 (nsp1, clone 12A4) directly conjugated with Alexa Fluor 488. A goat anti-mouse IgG1 conjugated to R-phycoerythrin (R-PE) was used as a secondary antibody for the anti-equine CD3 (Southern Biotech, Birmingham, AL).

**Table 5:** Monoclonal and polyclonal antibodies used for immunohistochemical and immunofluorescence staining.

Specificity	Clone	Species, isotype	Retrieval method	Source
CD8	CVS8	Mouse, IgG1	NA	AbD Serotec
CD4	CVS4	Mouse, IgG1	NA	AbD Serotec
CTLA-4	BNI3	Mouse, IgG2b	NA	Beckman-Coulter
Granzyme B	NA	Rabbit, polyclonal	Citrate-based (pH 6.0), 20 min	Spring Biosciences
Phosphorylated Akt	LP18	Mouse, IgG2b	Citrate-based (pH 6.0), 20 min	Leica Biosystems
NFATC2	25A10-D6-D2	Mouse, IgG1	Citrate-based (pH 6.0), 20 min	ThermoFisher Scientific
EOMES	WD1928	Mouse, IgG1	Citrate-based (pH 6.0), 20 min	ThermoFisher Scientific
PRDM1 (BLIMP-1)	NA	Rabbit, polyclonal	Citrate-based (pH 6.0), 20 min	ThermoFisher Scientific
TBX21 (T-bet)	NA	Rabbit, polyclonal	Citrate-based (pH 6.0), 20 min	ThermoFisher Scientific
Androgen receptor	NA	Rabbit, polyclonal	EDTA-based (pH 9.0), 20 min	Santa Cruz Biotechnologies
Ki-67	MIB-1	Mouse, IgG1	EDTA-based (pH 9.0), 20 min	Dako

Antigen retrieval (heat-induced epitope retrieval [HIER]) was performed on the BOND-MAX (Leica Biosystems). NA, not applicable.

### *CXCL16* genotyping

Stallions were genotyped based on the presence of 4 single nucleotide polymorphisms (SNPs) located in the *CXCL16* gene (*CXCL16S* and *CXCL16R*) by Sanger sequencing as previously described [29] and by the use of an allelic discrimination TaqMan real-time PCR (Table 1).

### CD3^+^ T lymphocyte phenotyping by dual-color flow cytometry

Peripheral blood mononuclear cells (PBMCs) were obtained by gradient centrifugation using Ficoll-Paque PLUS (GE Healthcare, Little Chalfont, UK) as previously described [31]. A total of 1x10^7^ PBMCs were infected with the VBS strain of EAV at a multiplicity of infection (MOI) of 2 for 36 h as described and subsequently dual stained for flow cytometric analysis [31]. Mock-infected PBMCs were used as negative controls and cultured under identical conditions. A total of 1x10^6^ cells were stained with anti-equine CD3 for 30 min followed by an R-PE conjugated goat anti-mouse IgG1 and subjected to intracellular anti-EAV staining using an anti-nsp1 antibody conjugated to Alexa Fluor 488 as previously described [31]. Acquisition was performed on a FACScalibur (BD, San Jose, CA). Lymphocytes were gated based on forward and side scatter parameters, and analyzed by two-color plots of anti-EAV nsp1 (FL-1) versus CD3 surface antigen (FL-2). The percentage of dual-positive (CD3^+^nsp1^+^) cells was determined by quadrant statistics using BD CellQuest (Table 1).

### Total RNA isolation for whole transcriptome sequencing (RNAseq)

Total RNA was isolated from cryosections of ampullae (naïve stallions, n = 3; short-term carrier, n = 6 and long-term carrier stallions, n = 3). Briefly, ten μm frozen sections (5 to 6) were collected into 1.5 ml tubes using a cryostat (Leica Biosystems) and immediately placed on dry ice. Subsequently, total RNA was isolated using an RNeasy Micro kit (Qiagen) following the manufacturer’s recommendations, and included an on-column DNase treatment. RNA yield was determined by fluorometry (Qubit RNA HS Assay kit, ThermoFisher Scientific) and quality was assessed using an Agilent RNA 6000 Pico kit (Agilent Technologies, Inc.) according to the manufacturer’s instructions.

### Whole transcriptome library preparation and Next Generation Sequencing (NGS)

Isolated RNA was submitted to Exiqon Services (Vedbæk, Denmark) and Qiagen Genomic Services (Hilden, Germany) for library preparation and sequencing, respectively. Library preparation was performed using the TruSeq stranded total RNA sample preparation kit with rRNA depletion (Illumina Inc., San Diego, CA). Libraries for each batch were pooled in equimolar ratios and sequenced on two High Output NextSeq500 runs with 2x50bp paired-end read length (2x30 million reads/sample) plus 2x8bp for demultiplexing. After sequencing, raw data were demultiplexed using the bcl2fastq v. 2.17 software (Illumina Inc.). FastQ for each of the two were combined and QC was performed using the FastQC software package v. 0.10.1 (Babraham Bioinformatics, Babraham Institute, Cambridge, UK).

### Whole transcriptome sequencing data analysis

Adaptor trimming and quality control were performed using TrimGalore Version 0.4.4 (Babraham Bioinformatics) and reads were subsequently aligned to the *Equus caballus* reference genome (EquCab2.0) [66] using Burrows-Wheeler Aligner (bwa; Version 0.7.12) [67]. Reads were annotated to the equine reference transcriptome available in the Ensembl database (EquCab2.88; www.ensembl.org) using Cufflinks (Release 2.2.1) [68]. Read counts were normalized as fragments per kilobase of exon per million mapped reads (FPKM) and differential gene expression analysis was performed on normalized read counts. Cuffdiff was used to perform differential gene expression analysis between experimental groups (pairwise comparisons): naïve vs long-term carrier stallions, naïve vs short-term carrier stallions, long-term carrier stallions vs short-term carrier stallions and *CXCL16S* (homozygous and heterozygous) stallions vs *CXCL16R* (homozygous) stallions [68]. Significance was established if the false discovery rate (FDR) was <0.1. DEGs were defined by FDR < 0.1 and log_2_ fold-change > 1 (upregulated) or < 1 (downregulated). Mapping statistics are shown in S8 Table.

### Functional annotation, pathway analysis and upstream regulator analysis

To investigate the molecular and biological functions of differentially expressed genes and those derived from modular analysis, DAVID Bioinformatics Resources (version 6.8 [https://david.ncifcrf.gov/], National Institute of Allergy and Infectious Disease [NIAID], National Institutes of Health [NIH]) [33, 69] along with the PANTHER classification system (www.pantherdb.org) [32] were used to functionally annotate genes based on gene ontology (biological process, molecular function and protein class). Ingenuity Pathway Analysis (IPA, Qiagen) was used to perform canonical pathway analysis, upstream regulator analysis with a special emphasis on transcription factors and identification of the most significant molecular networks.

### Transcription factor binding site analysis

Differentially expressed genes between long-term and short-term carrier stallions were analyzed using a recently developed transcription factor analysis software (CiiiDER, Centre for Innate Immunity and Infectious Diseases, Hudson Institute of Medical Research, Victoria, Australia) [35]. CiiiDER can identify over-represented transcription factors that may be potentially playing an important role in regulating the genes of interest by comparing the predicted binding sites present in a list of co-regulated genes to those found in an appropriate background list of genes. A background gene list was generated by selecting genes that were not differentially expressed (FDR > 0.1) and had a very low fold-change (log_2_ fold-change between -1 and 1) as recommended. Enrichment analysis was performed as suggested and a Fisher’s exact test built-in the algorithm was used to identify significantly over-represented (enriched) transcription factors [35].

### Total RNA isolation for reverse transcription quantitative real-time PCR

Additionally, total RNA was isolated from ampullae for RT-qPCR analysis using the RNeasy Mini kit (Qiagen) according to the manufacturer’s recommendations as previously described [30], including on-column DNase treatment.

### Quantitative real-time PCR for gene expression analysis

Expression levels of selected genes were determined by RT-qPCR (S9 Table). Total RNA was reverse transcribed using the High Capacity cDNA Reverse Transcription kit (ThermoFisher Scientific) following the manufacturer’s recommendations. The reaction (20 μl) included 10 μl of total RNA (2 μg), 2 μl of 10X RT Buffer, 0.8 μl 25X dNTP Mix (100 mM), 2 μl of 10X RT Random Primers, 1 μl of MultiScribe Reverse Transcriptase, 1 μl of RNase inhibitor and 3.2 μl of nuclease-free water. The reverse transcription reaction was incubated for 10 min at 25°C, followed by 120 min incubation at 37°C and a final step at 85°C for 5 min. cDNA was diluted 1:5 in nuclease-free water and stored at -20°C until used. For qPCR, the PowerUp SYBR Green Master Mix (ThermoFisher Scientific) was used. Briefly, 1 μl of cDNA was added to a reaction volume (9 μl) containing 2X PowerUp SYBR Green Master Mix (5 μl), assay-specific forward and reverse primers (0.25 μl of a 20 μM stock, final concentration 0.5 μM for each primer) and RNase-free water (3.5 μl). The cycling conditions included an initial UDG (50°C for 2 min) and PCR activation steps (95°C for 2 min) followed by 40 cycles of denaturation at 95°C for 15 s and annealing/extension at 60°C for 1 min. Melt curve analysis was performed to check for non-specific amplifications along with the inclusion of non-template controls. Specific forward and reverse primers were designed using the Primer-BLAST tool (NCBI, NIH) and primer sequences and gene accession numbers are shown in S9 Table. qPCR efficiencies and Ct values were determined using LinRegPCR v2017.0 [70], with efficiencies in the range of 90.2–100.2%. qPCR reactions were performed in duplicate and Ct values >37 were not used for analysis. Gene expression data were normalized to three housekeeping genes (*GAPDH*, *ACTB*, and *GUSB*) as previously described [30].

### *CXCL16* and *CXCR6* quantitative TaqMan real-time PCR

*CXCL16* and *CXCR6* expression was evaluated by TaqMan quantitative real-time PCR. Reverse transcription was performed as indicated above. Complementary DNA was diluted 1:1 in nuclease-free water and stored at -20°C until used. A custom TaqMan Gene Expression assay for equine *CXCL16* was used as previously described [30]. In addition, a custom TaqMan Gene Expression assay for equine *CXCR6* was developed by a commercial company (ThermoFisher Scientific) using the mRNA sequences derived from GenBank accession number XM_005600758.3. The qPCR reaction and cycling conditions were performed as previously described [30]. RT-qPCR efficiencies, normalization and analysis were performed as indicated above.

### *CXCL16* and *CXCR6*-specific *in situ* hybridization (ISH [RNAscope])

A *CXCL16* mRNA-specific probe based on the GenBank accession number XM_001504756.5 was developed by a commercial company (Advanced Cell Diagnostics [ACD], Newark, CA) as previously described [30]. A *CXCR6* mRNA-specific probe based on the GenBank accession number XM_005600758.3 was developed, containing a total of 20 double Z branched DNA probe pairs spanning a target region of 874 bp of the mRNA sequence (nt position 154–1028). The probes were supplied in a ready-to-use format, and their specificity was evaluated using lymphoid tissues (palatine tonsils; University of Kentucky, Lexington, KY), an equine endothelial cell line known to abundantly express *CXCL16* as previously described [30] and a stable cell line expressing equine *CXCR6* (HEK-CXCR6; University of Kentucky, Lexington, KY) [29]. Probes specific to dihydrodipicolinate reductase B mRNA of *Bacillus subtilis* and peptidylprolyl isomerase B (PPIB) were used as negative and positive controls to assess assay specificity and RNA integrity in FFPE tissues, respectively. The RNAscope ISH assay was performed using the RNAscope 2.0 HD Red Detection Kit (ACD, Hayward, CA) as previously described [30]. For *CXCL16* mRNA-specific ISH, signal in the region of interest was quantified as previously indicated [30]: (0), no staining or <1 dot every 10 cells at 40X magnification; (1), 1–3 dots every 10 cells at 40X magnification; (2), 4–10 dots/cell (visible at 20-40X magnification); (3), >10 dots/cell with scattered cells presenting dot clusters (visible at 20X magnification); (4), >10 dots/cell with frequent cells presenting dot clusters (visible at 20X magnification). For CXCR6 mRNA-specific ISH, signal was quantified as follows: (0), no staining or rare positive (1 dot) cells at 40X magnification; (1), scattered positive cells (1–3 dots) at 40X magnification; (2), 1–3 dots/cell (visible at 20-40X magnification); (3), >3 dots/cell with scattered cells presenting dot clusters (visible at 20X magnification); (4), >3 dots/cell with frequent cells presenting dot clusters (visible at 20X magnification).

### Immunohistochemistry (IHC)

For IHC, cryosections (10 μm, only for CD8, CD4 and CTLA-4 antigens) or sections of FFPE tissues (5 μm) were mounted on positively charged Superfrost Plus slides (Fisher Scientific, Pittsburgh, PA) and processed as previously described [23]. Specific retrieval conditions for each antigen are shown in Table 5. For Ki-67, phosphorylated Akt, NFATC2, EOMES, BLIMP-1, T-bet, CD8, CD4, AR and granzyme B immunostaining was performed using the Bond Polymer Refine Detection kit (Leica Biosystems, Buffalo Grove, IL). The slides were incubated with 3% hydrogen peroxide (5 min), followed by incubation with the primary antibody diluted in ISH/IHC Super Blocking (Leica Biosystems) for 1 h at room temperature. This was followed by incubation with a rabbit anti-mouse IgG (8 min) followed by a polymer-labeled goat anti-rabbit IgG conjugated to HRP (8 min) in the case of mouse primary antibodies. In the case of primary antibodies of rabbit origin, tissue sections were directly incubated with the polymer-labeled goat anti-rabbit IgG conjugated to HRP after primary antibody incubation. 3,3'-diaminobenzidine tetrahydrochloride (DAB) was used as the substrate and the slides were incubated for 10 min. For NFATC2 and granzyme B immunostaining, sections were incubated with a ready-to-use copper sulfate solution (DAB Enhancer, Leica Biosystems) to enhance the signal. Finally, sections were counterstained with hematoxylin and mounted as previously described [23, 24, 71]. For CTLA-4, the Bond Polymer Refine Red Detection kit (Leica Biosystems) was used. After incubation with the primary antibody (1h at room temperature), tissue sections were incubated with a rabbit anti-mouse IgG (20 min) followed by a polymer-labeled goat anti-rabbit IgG coupled with alkaline phosphatase (AP, 30 min). Fast Red was used as the chromogen (15 min), and counterstaining and mounting was performed as indicated above. Palatine tonsil sections were used as both positive and negative (irrelevant primary antibody) controls (S5 Fig). Immunostaining was semi-quantitatively scored based on the cumulative number of positive cells in five high magnification (40X) microscopic fields (Table 6). To quantify and compare the predominance of specific transcription factors in inflammatory infiltrates present in long-term carrier stallions, the number of positive cells in five specific inflammatory infiltrates were counted at 100X magnification. Proliferation was assessed specifically on epithelial cells and infiltrating lymphocytes by means of Ki-67 immunostaining [34].

**Table 6:** Scoring system used for immunohistochemical staining of specific cellular markers. Numerical values express the cumulative number of immunohistochemical positive cells in five microscopic fields (400X).

Score	AR, EOMES, NFATC2, PRDM1, TBX21, pAkt	Granzyme B, CTLA-4
**0**	None	None
**1**	<50	<10
**2**	50–100	11–20
**3**	101–200	21–30
**4**	>200	>30

### Immunofluorescence

Snap-frozen tissue sections (10 μm) were obtained and stained for CD8 as previously described [23]. Following fixation, permeabilization and blocking, sections were incubated with mouse anti-equine CD8 for 1 h at room temperature in a humidity tray (Table 5), followed by a F(ab')2 fragment of goat anti-mouse IgG conjugated with Alexa Fluor 488 (Life Technologies) diluted 1:200 in 5% normal goat serum (Jackson ImmunoResearch, West Grove, PA) for 1 h at room temperature in a humidity tray. Nuclear counterstaining was performed with a mounting medium containing 4',6-diamidino-2-phenylindole (DAPI; Vector Laboratories, Burlingame, CA) and observed under a Nikon Ti fluorescent microscope (Nikon Corporate, Tokyo, Japan).

### Weighted gene co-expression network construction, module identification, hub gene selection and visual network representation

Weighted gene co-expression network analysis was used to construct co-expression networks using the R package “WGCNA” (Department of Human Genetics and Department of Bioinformatics, University of California Los Angeles, Los Angeles, CA) as previously described and following the steps shown under WGCNA tutorials [45]. Gene co-expression networks were generated for the whole transcriptome of *CXCL16S* and *CXCL16R* stallions (n = 6 per group) and TFs in *CXCL16S* stallions based on their gene expression profiles. A total of 12,303 genes were selected based on expression in at least 50% of the samples. Following construction of a matrix of pairwise correlations between all pairs of genes across the samples, a weighted adjacency matrix was generated by raising co-expression similarity to a power β = 16 as determined for this sample set. Subsequently, a topological overlap matrix (TOM) was constructed and used as input for hierarchical clustering analysis. Gene modules (i.e. genes with high topological overlap) were identified using a dynamic tree cutting algorithm (deepSplit = 2, cutHeight = 0.25) and visualized by heatmap plot of the topological overlap of the gene network. Module relationships were summarized by a hierarchical clustering dendrogram and heatmap plot of module eigengenes. These steps were performed using the automatic network construction and module detection functions with default parameters except maxBlockSize = 12,500 and minModuleSize = 30. The associations between the gene modules and the trait of interest (percentage of CD3^+^ T lymphocytes susceptible to in vitro EAV infection/*CXCL16* genotype) were tested by correlating MEs to trait measurements. Module-trait associations were visualized using a heatmap plot. Gene ontology analysis (biological process) was performed on gene lists derived from selected modules (r > 0.5 and *p*-value < 0.05) using DAVID as indicated above. Module memberships (MM, i.e. the correlation of each gene’s expression value with the module eigengene of a given module as a measure of the intramodular connectivity) and gene significance (GS, i.e. the correlation between the gene expression profile and the trait as a measure of biological relevance) were calculated [45, 47]. Modules correlated with the trait were analyzed using the NetworkAnalysis tool in Cytoscape (version 3.1.0) [72]. The genes (network nodes) having MM ≥ 0.90, *p*-value < 0.05, and GS ≥ 0.5 were identified as intramodular hub genes [73]. The gene co-expression networks for selected modules of genes were visualized using Cytoscape.

### Statistical analysis

Data distribution, descriptive statistics, plots, and statistical tests were generated using JMP13 Pro statistical analysis software (Cary, NC). Heatmaps were built using Package 'd3heatmap' in R. Whole transcriptome data was analyzed as indicated above (see Whole transcriptome sequencing data analysis). RT-qPCR data (-ΔCt values) were analyzed using a one-way analysis of variance (ANOVA; JMP13 Pro) and post-hoc comparisons were performed using the Student’s *t-*test [74]. Statistical significance was set at a *p*-value < 0.05. Finally, RNAscope ISH and IHC data were subjected to a non-parametric test (Kruskal-Wallis) using JMP13 Pro and statistical significance was set at a *p*-value <0.05. To evaluate the predominance of specific TFs in inflammatory infiltrates during long-term persistence, the average number of positive cells among the long-term persistently infected stallions (n = 3) was calculated and analyzed using a one-way ANOVA and post-hoc comparisons were performed using the Student’s *t-*test. Statistical significance was set at a *p*-value < 0.05.

### Accession numbers

The RNA sequencing data from this study were deposited in the Gene Expression Omnibus (GEO, NCBI, NIH) database under study GSE114982 (accession numbers GSM3161940- GSM3161951).

## Supporting information

S1 Fig Gene ontology analysis of DEGs between long-term carrier (n = 3) and short-term carrier stallions (n = 6). (A) Biological process. (B) Molecular function.
(TIF)

S2 Fig Inflammatory response in the ampullae during long-term EAV persistence. (A and inset) The inflammatory response was characterized by moderate to severe, multifocal lymphoplasmacytic ampullitis. H&E. 50X. Bar = 300 μm. (B) The inflammatory infiltration is characterized by the predominance of CD8^+^ T lymphocytes. CD8-specific immunostaining. DAB. 100X. Bar = 80 μm. (C) The lymphocytic infiltration was also closely associated with the luminal epithelium, with presence of intra- and subepithelial CD8^+^ T lymphocytes (inset [arrowheads], CD8-specific immunofluorescence [green]). H&E. 400X. Bar = 20 μm.
(TIF)

S3 Fig Gene ontology analysis (biological process) of DEGs between stallions homozygous and heterozygous for *CXCL16S* (n = 5) and stallions homozygous for *CXCL16R* (n = 4). (TIF)

S4 Fig Hierarchical clustering of module eigengenes (MEs) for the modules identified. (A) Hierarchical clustering of MEs for the 24 modules identified. Branches of the dendrogram group together MEs that are positively correlated. The *blue* and *lightyellow* modules are positively correlated with the trait (percentage of CD3^+^ T lymphocytes susceptible to *in vitro* EAV infection*). (B) ME adjacency heatmap depicting correlation among modules. (C) Hierarchical clustering of MEs corresponding to a transcription factor-specific network construction (n = 494 transcription factor genes) revealed that the over-represented transcription factors associated with EAV persistence clustered within a single module (*green*).
(TIF)

S5 Fig Immunohistochemical staining for EOMES, NFATC2, TBX21 (T-bet), BLIMP-1, pAkt, CTLA-4 and granzyme B on equine palatine tonsil (tissue control). The negative immunostaining control is labeled as isotype control. DAB. 100X. Bar = 80 μm.
(TIF)

S1 Table Top 25 canonical pathways associated with common upregulated genes. Pathway analysis was performed using IPA.
(XLSX)

S2 Table Transcription factors (TFs) identified by means of upstream regulator analysis performed on Ingenuity Pathway Analysis (IPA) in long-term carrier stallions. Target molecules in dataset are depicted.
(XLSX)

S3 Table Target and correlated genes obtained from the Ingenuity Knowledgebase (IPA), the Immuno-Navigator database and literature search associated with (A) EOMES, (B) NFATC2, (C) TBX21 and (D) PRDM1.
(XLSX)

S4 Table Genes positively correlated to CXCL16 and CXCR6 (*R^2^* greater or equal to 0.5) and differentially expressed in long-term carrier stallions. Correlated genes were identified using the immune database Immuno-Navigator and intersected with the differentially expressed genes identified in the long-term carrier stallions (n = 3) compared to short-term carrier stallions (n = 6).
(XLSX)

S5 Table Gene members of modules correlated with the CD3+ T lymphocyte susceptibility phenotype/CXCL16 genotype (*blue* and *lightyellow*) following weighted gene co-expression network analysis of 12,303 genes. (XLSX)

S6 Table Network analysis parameters for the "hub" genes in the *blue* (n = 1135 total genes) and *lightyellow* (n = 130 total genes) modules (MM≥0.90, p-value<0.05, GS≥0.5). Network analysis was performed using Cytoscape.
(XLSX)

S7 Table Gene members in each module (n = 5 modules) following weighted gene co-expression analysis of 494 transcription factor genes in *CXCL16S* stallions. (XLSX)

S8 Table RNA sequencing mapping statistics. Whole transcriptome sequencing was performed on High Output NextSeq500 runs with 2x50bp paired-end reads. After sequencing, raw data were demultiplexed using the bcl2fastq v. 2.17 software (Illumina Inc.), quality assessed using the FastQC software package v0.10.1, trimmed using TrimGalore v0.4.4 and mapped to the reference *Equus caballus* genome (EquCab2.1) using Burrows-Wheeler Aligner (bwa v.0.7.12).
(XLSX)

S9 Table Primers designed for gene expression analysis by RT-qPCR. (DOCX)
