# Supplementary figures and images for: Equine arteritis virus long-term persistence is orchestrated by CD8+ T lymphocyte transcription factors, inhibitory receptors, and the CXCL16/CXCR6 axis
Source: PLoS Pathog. 2019 Jul 29;15(7):e1007950. doi: 10.1371/journal.ppat.1007950 (PMC6692045; doi:10.1371/journal.ppat.1007950)

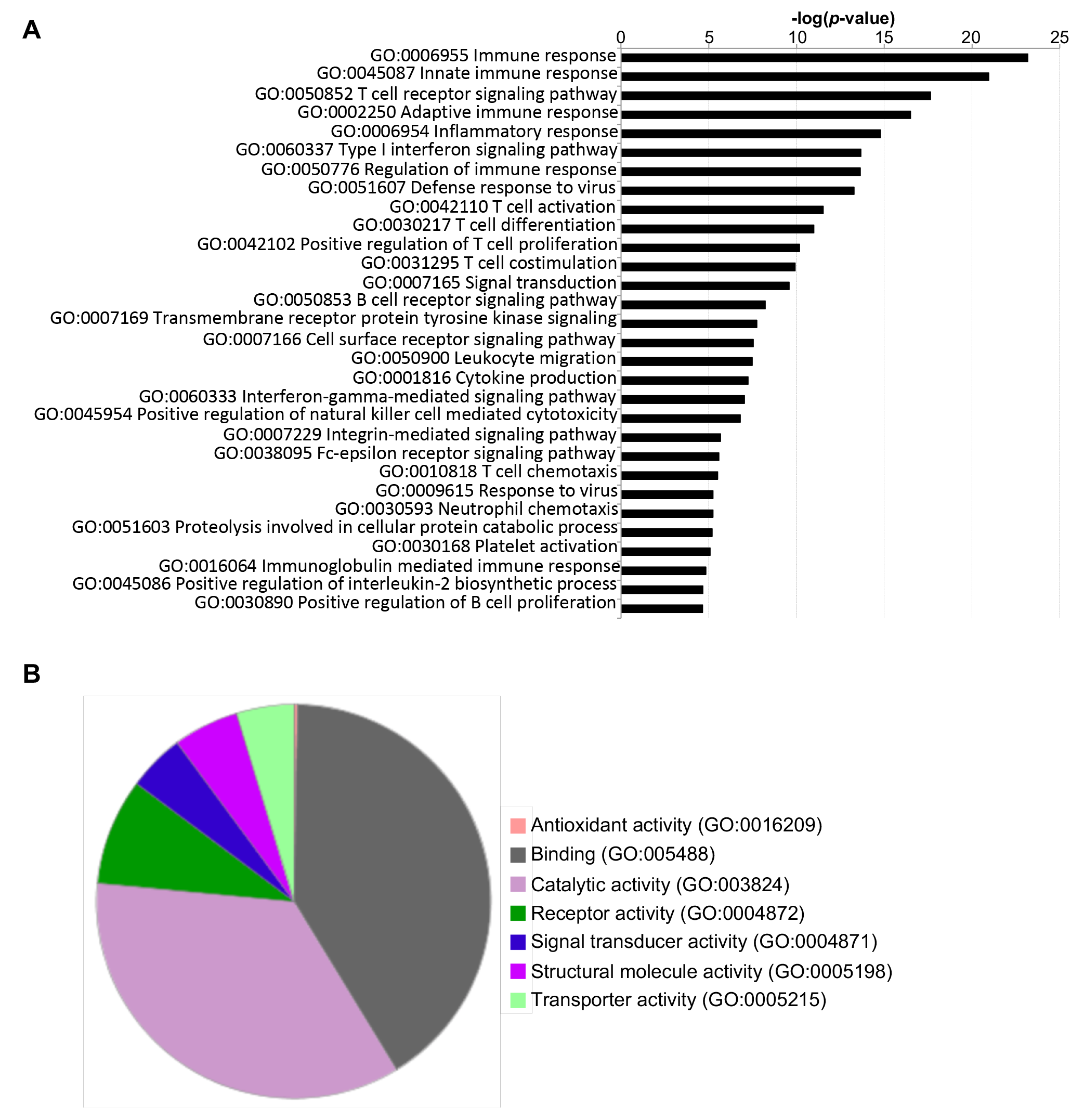

Supplement: S1 Fig — (A) Biological process. (B) Molecular function. (TIF) [file ppat.1007950.s001.tif]

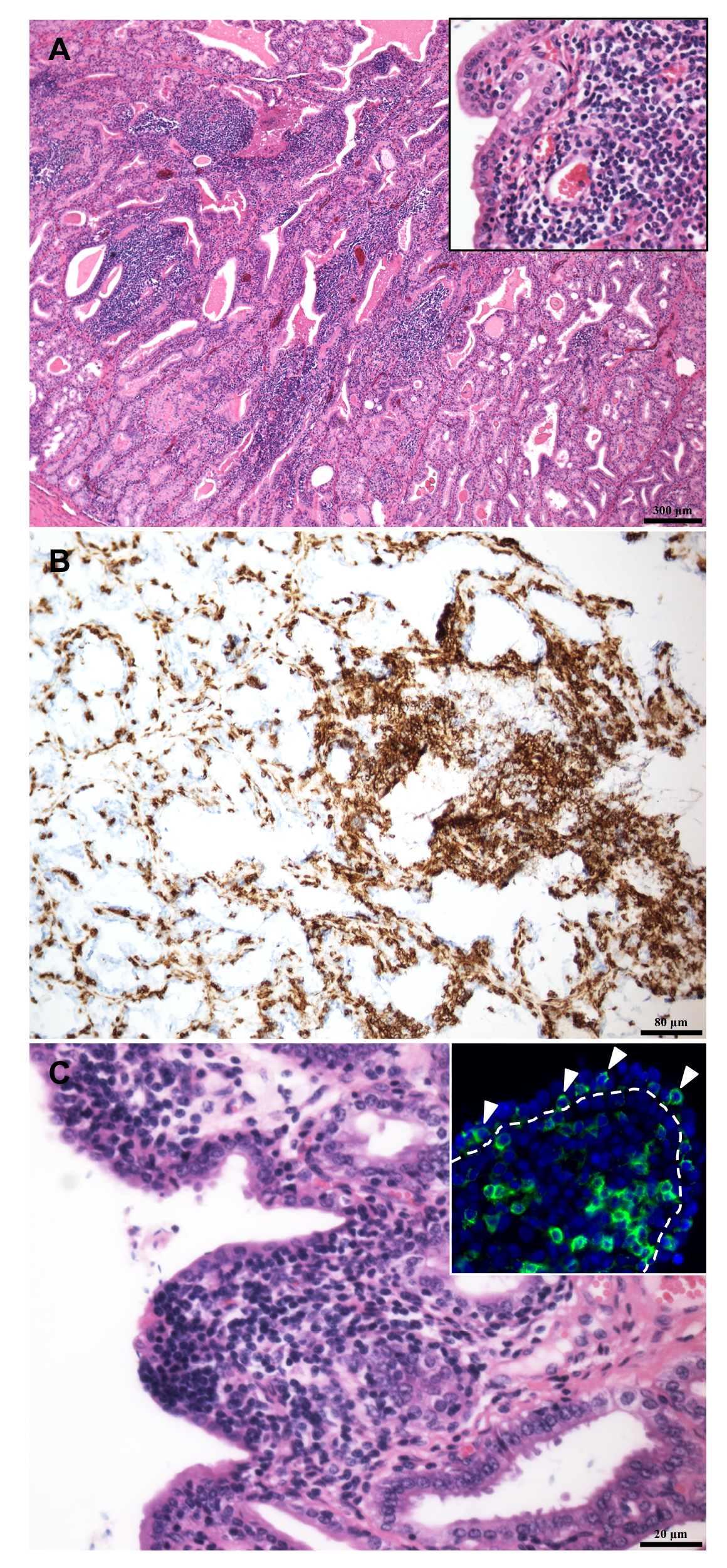

Supplement: S2 Fig — (A and inset) The inflammatory response was characterized by moderate to severe, multifocal lymphoplasmacytic ampullitis. H&E. 50X. Bar = 300 μm. (B) The inflammatory infiltration is characterized by the predominance of CD8+ T lymphocytes. CD8-specific immunostaining. DAB. 100X. Bar = 80 μm. (C) The lymphocytic infiltration was also closely associated with the luminal epithelium, with presence of intra- and subepithelial CD8+ T lymphocytes (inset [arrowheads], CD8-specific immunofluorescence [green]). H&E. 400X. Bar = 20 μm. (TIF) [file ppat.1007950.s002.tif]

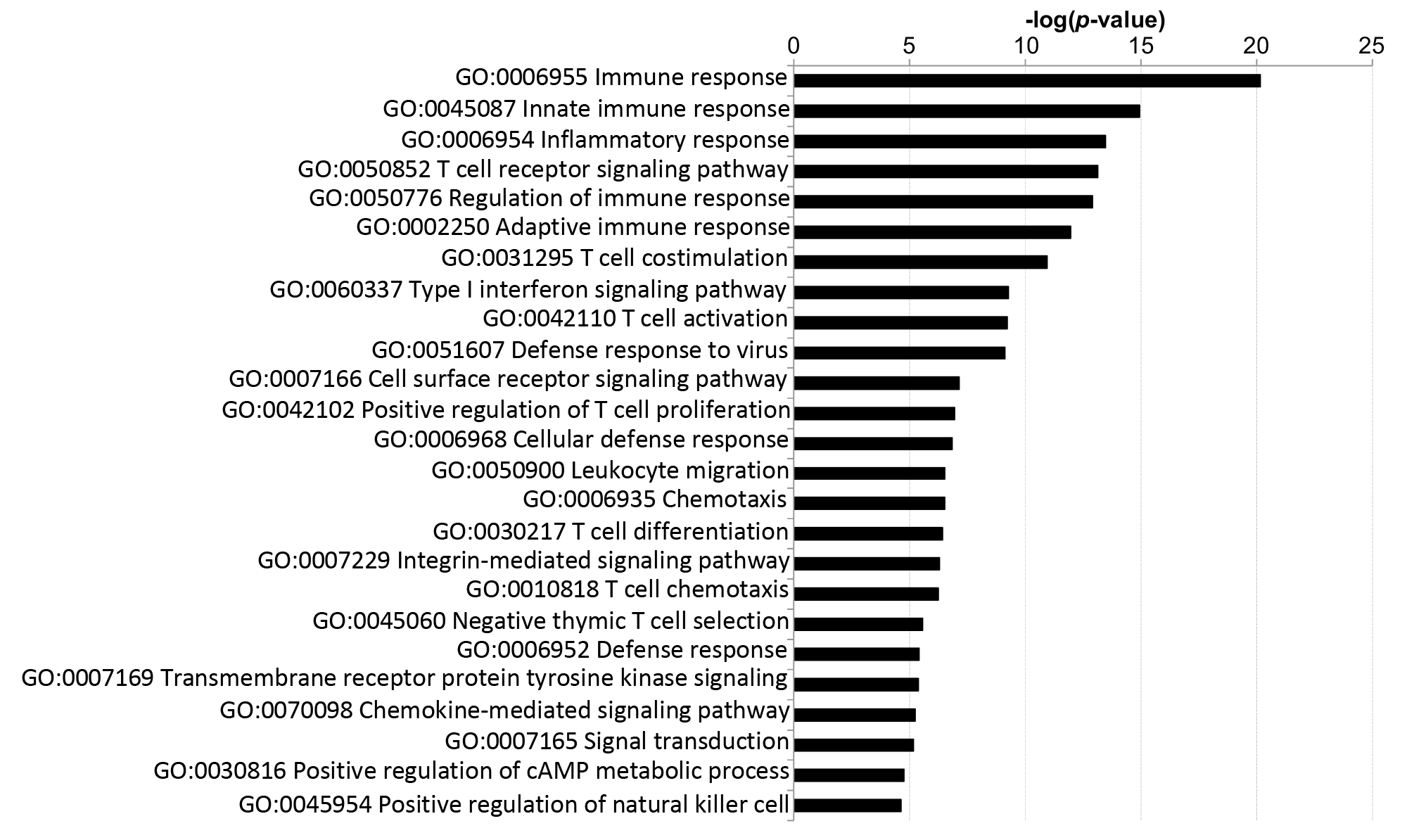

Supplement: S3 Fig — (TIF) [file ppat.1007950.s003.tif]

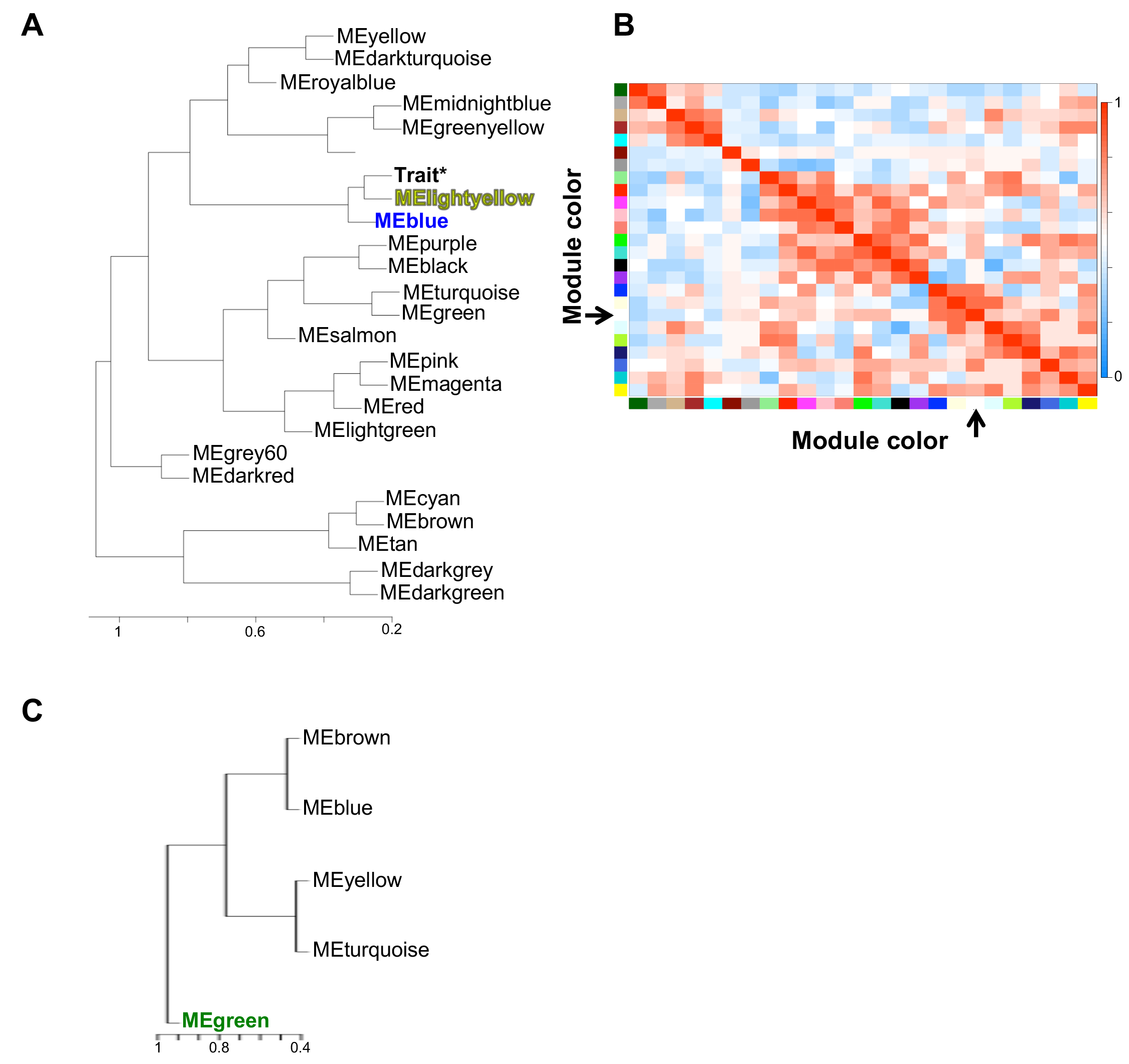

Supplement: S4 Fig — (A) Hierarchical clustering of MEs for the 24 modules identified. Branches of the dendrogram group together MEs that are positively correlated. The blue and lightyellow modules are positively correlated with the trait (percentage of CD3+ T lymphocytes susceptible to in vitro EAV infection*). (B) ME adjacency heatmap depicting correlation among modules. (C) Hierarchical clustering of MEs corresponding to a transcription factor-specific network construction (n = 494 transcription factor genes) revealed that the over-represented transcription factors associated with EAV persistence clustered within a single module (green). (TIF) [file ppat.1007950.s004.tif]

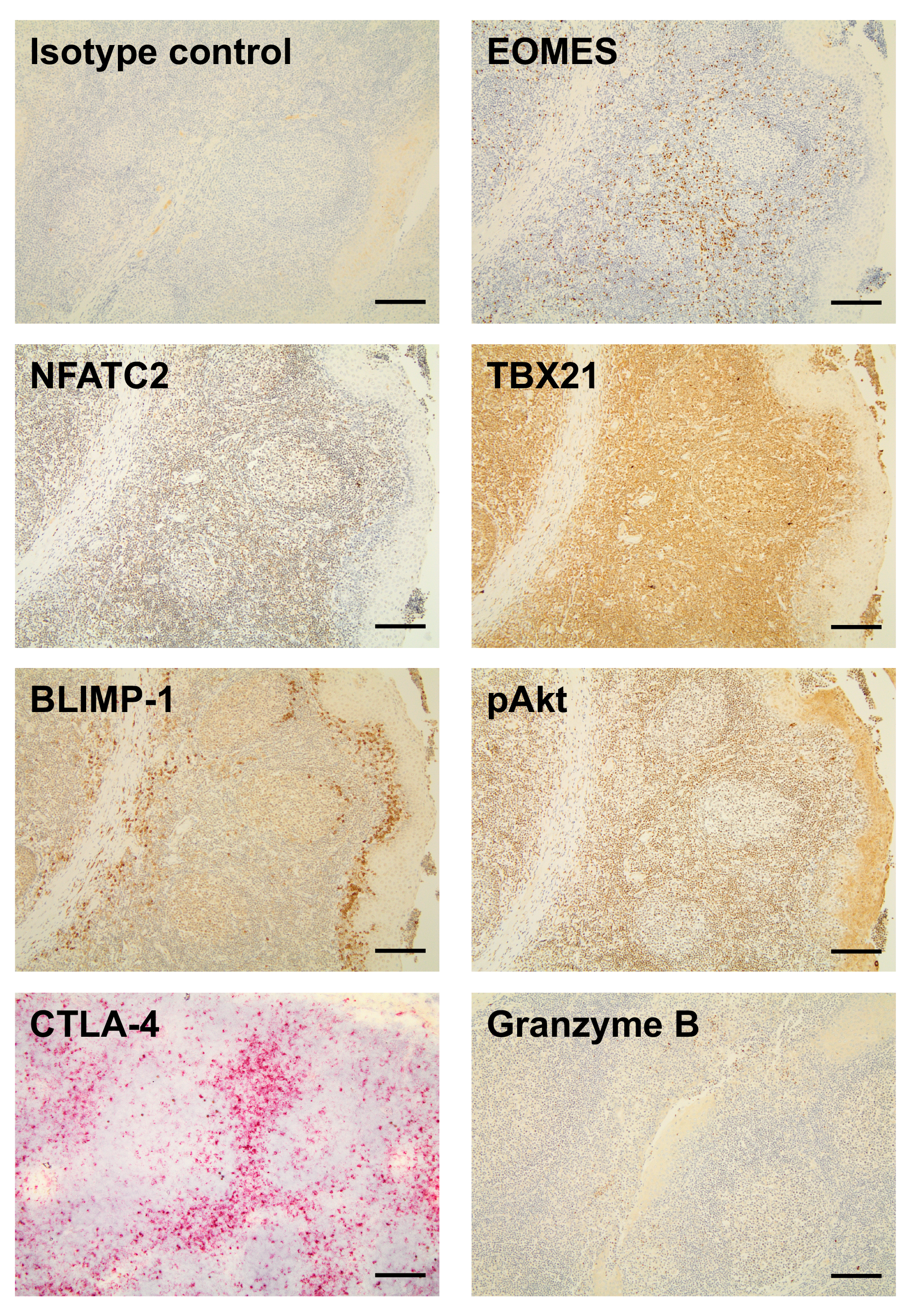

Supplement: S5 Fig — The negative immunostaining control is labeled as isotype control. DAB. 100X. Bar = 80 μm. (TIF) [file ppat.1007950.s005.tif]
