# Supplementary material for: Equine arteritis virus long-term persistence is orchestrated by CD8+ T lymphocyte transcription factors, inhibitory receptors, and the CXCL16/CXCR6 axis
Source: PLoS Pathog. 2019 Jul 29;15(7):e1007950. doi: 10.1371/journal.ppat.1007950 (PMC6692045; doi:10.1371/journal.ppat.1007950)
Supplement: S9 Table — (DOCX) [file ppat.1007950.s014.docx]

**S9 Table** Primers designed for gene expression analysis by RT-qPCR

| **Gene** | **Sequence (5**′ **to 3**′**)** | **Accession number** |
| --- | --- | --- |
| BATF | GACCTGGAGAAACAGAACGC (forward)  TGCTCAGCACTGATGTGAAGT(reverse) | XM_001492166.3 |
| EOMES | GGCGTGGAGGACTTGAATGA (forward)  TGTTTTGGTAGGCCGTCACT (reverse) | XM_001493356.3 |
| FOS | CCTGCAAGATCCCCGATGAC (forward)  CAGGATCGTTAAGGAGGGGC (reverse) | XM_001491972.4 |
| IKZF1 | TGATGAACCCATGCCTGTCC (forward)  CCACTCCTCTCTCACTCTTGG (reverse) | XM_023639052.1 |
| IRF1 | CTCTGATGGACTCAGCAGCTC (forward)  TCCAAGTCCTGCCCCATGTA (reverse) | XM_001504445.3 |
| IRF4 | TGCGATGCGCTTTGAACAAG (forward)  TTGGCTCCTTCGGGAACAAT (reverse) | XM_023624331.1 |
| JUNB | TTTCAGACCTCGGTAGGGGT(forward)  TACCACGACGACTCATACGC (reverse) | XM_014741462.2 |
| NFATC2 | TGCGAAGTTCTCGCAGTACA (forward)  CTCACCCGTGATCCACTACC (reverse) | XM_005604783.3 |
| NFKBIA | CAAAAGGAGGGCTGATCCCC (forward)  CTGACGCTATGAGCTTCGGA (reverse) | XM_023624022.1 |
| PRDM1 | ACTGGACTCCAACCCTTCCA (forward)  TAGACCACCCGTGGGTAGAA (reverse) | XM_005596848.3 |
| STAT1 | CGTGATAGGGTCGTGTTCGT (forward)  TCACCATAGTCGCGGAGAGT (reverse) | XM_001499369.5 |
| STAT2 | GTAATGCCCCCTTCCGATGT (forward)  ATCATGGGCTTTGTGAGCCG (reverse) | XM_001504841.3 |
| TBX21 | CGGCCTGCATTACCTCCAAA (forward)  TGGTAGGCAGTCACAGCAAT (reverse) | XM_023652657.1 |
| CCL5 | CCTACATCTCCCGCCCACTG (forward)  ATAGAGCACTTGCTGCTGGTGT (reverse) | NM_001081863.2 |
| CXCL9 | CTCCAGTAATGAGGAAGGGACG (forward)  AACAGTTTTGGGCGGATCGT (reverse) | XM_023636871.1 |
| CXCL10 | TGCACGCTGTACCTGCATTA (forward)  ACGTTGACAAGATTGACTTGCAG (reverse) | NM_001114940.1 |
| CXCL11 | GGCCCTGGAGTAAAAGCAGT (forward)  TTGTCACAGTTGTTACTTGGGT (reverse) | NM_001278930.2 |
| CXCR3 | TTGACACCCACAAAGGCGTA (forward)  GGATGTGGCCAAGTCTGTCA (reverse) | XM_001493611.6 |
| IFNG | ACGCGGCCTGGCAGTAATA (forward)  ACAAGTTTTATCTTGGCTTTTCAGC (reverse) | NM_001081949.1 |
| IL2 | TCAAACCTCTGGAGGAAATGCT (forward)  AGTCCCAGAACTGTTACATTGA (reverse) | NM_001085433.2 |
| TNFA | CGTCCAAGGTCAACCTCCTC (forward)  TGGGCTGATTGATCTCAGCG (reverse) | XM_005603490.1 |
| **Gene** | **Sequence (5**′ **to 3**′**)** | **Accession number** |
| CD160 | TGGCACAACACTGGTAGGTC (forward)  TTTTGTGCAAGGACAGGGGT (reverse) | XM_005610210.3 |
| CD244 | TCGACGAACCTCACCTACCT (forward)  GATGCCCAGCTGACAGGATT (reverse) | XM_023640876.1 |
| CD274 | CCCGAAGGTTCAGCACAGTA (forward)  TGATCTGAAGTGCGGCCTTT (reverse) | XM_001492842.6 |
| CD40LG | AGGTCCGTGAGTGGATCTGA (forward)  GACAAACACCGAAGCACCTG (reverse) | XM_001490011.5 |
| CD69 | TTTGGGCCAAGGTCCAGTTT (forward)  TGTCCAGGCCAACATACGTC (reverse) | XM_001499388.4 |
| CTLA4 | AGAAGTCCTCTTACTACAGGGGT (forward)  AAGGCTGAAATTGCTTTTCACA (reverse) | XM_023622472.1 |
| FASLG | TCCAACCCTCTGGAATGGGA (forward)  AGTACAGCCCGGTGTCATTG (reverse) | NM_001166039.1 |
| HAVCR2 | AAGGTGGGTCAGAATGCACA (forward)  CAGCACACAGGCACGAGAT (reverse) | XM_005599264.3 |
| LAG3 | CAGCTTCTGTGCACTGGTTC (forward)  CCGGTGAAGTAGTGATGGGG (reverse) | XM_001492398.5 |
| PDCD1 | CGAGGGCTCTCGTTGATCTG (forward) GACTTCCACATGAGCGTCCT (reverse) | XM_023642815.1 |
| SEMA7A | CCGATACCCACAGAGACCTTC (forward)  GTGGACGACCACTTTCTGGTA (reverse) | XM_001917675.4 |
| CD44 | GTTAACCGTGATGGCACTCG (forward)  GGTGCTGGGGTTGATGTCTT (reverse) | XM_005598012.3 |
| DGKA | CAATCACATGTGTGGGTGCG (forward)  GCCCAACTAGGCTGTGGTAG (reverse) | XM_023643757.1 |
| ACTB | CGACATCCGTAAGGACCTGT (forward)  CAGGGCTGTGATCTCCTTCT (reverse) | NM_01081838 |
| GAPDH | AGAAGGAGAAAGGCCCTCAG (forward)  GGAAACTGTGGAGGTCAGGA (reverse) | NM_001163856.1 |
| GUSB | GGGATTCGCACTGTGGCTGTCA (forward)  CCAGTCAAAGCCCTTCCCTCGGA (reverse) | XM_014729943.1 |
